# Supplementary material for: Genome-wide association study of body fat distribution identifies adiposity loci and sex-specific genetic effects
Source: Nat Commun. 2019 Jan 21;10:339. doi: 10.1038/s41467-018-08000-4 (PMC6341104; doi:10.1038/s41467-018-08000-4)
Supplement: Supplementary file 1 — Supplementary Information [file 41467_2018_8000_MOESM1_ESM.pdf]

Genome-Wide Association Study of Body Fat Distribution identifies Adiposity Loci and Sex-Specific Genetic Effects

Supplementary Figures and Tables

Rask-Andersen M et al.

Table of contents:

Supplementary Table 1. Basic characteristics of UK Biobank participants included in the analyses.
Supplementary Table 2. SNP-heritability (*h2*) and LD score regression intercept estimates of body fat ratios.
Supplementary Figure 1. Quantile-quantile (QQ) plots of the distribution of P-values from GWAS of body fat ratios in the UK Biobank sex-combined, female and male discovery cohorts.
Supplementary Figure 2. Manhattan plots for GWAS results for arm fat ratio in the combined discovery cohort and sex-stratified analyses.
Supplementary Figure 3. Manhattan plots for GWAS results for leg fat ratio in the combined discovery cohort and sex-stratified analyses.
Supplementary Figure 4. Manhattan plots for GWAS results for trunk fat ratio in the combined discovery cohort and sex-stratified analyses.
Supplementary Table 3. Independent signals* for association with body fat ratios in the combined cohort.
Supplementary Table 4. Independent signals for association with body fat ratios in females.
Supplementary Table 5. Independent signals for association with body fat ratios in males.
Supplementary Table 6. Direction of effect for body fat ratio–associated lead SNPs for standard anthropometric traits.
Supplementary Table 7. Overlap between body fat ratio-associated loci with non-anthropometric trait-associations from previous GWAS.
Supplementary Table 8. Phenotypic correlation between body fat ratios and anthropometric traits.
Supplementary Table 9. Genetic correlations between body fat ratios and anthropometric traits.
Supplementary Table 10. Body fat ratio-associated potentially damaging missense variants.
Supplementary Figure 5. Overlap of enriched gene sets from DEPICT for body fat ratios.
Supplementary Figure 6. Flowchart of the genetic analyses of body-fat ratios.
References

Supplementary Table 1. Basic characteristics of UK Biobank participants included in the analyses. Data is presented for the discovery and replication cohorts. Mean values are presented ± standard deviations. P-values denote results from student's t-tests for differences between females and males in the discovery and replication cohort, as well as differences between the replication and discovery cohorts. Bonferroni adjustment for multiple testing gives that $P < 0.05/42$ can be considered significant.

	**Discovery cohort**		**Differences males/females (*P*)**	**Replication cohort**		**Differences males/females (*P*)**	**Differences between discovery and replication (P)**	
	**Males**	**Females**		**Males**	**Females**		**Males**	**Females**
N	55,006	61,132		112,425	133,936			
Age (years)	57.5 ± 8.1	56.8 ± 7.9	$<2.2*10^{-16}$	57.1 ± 8.1	56.7 ± 7.9	$<2.2*10^{-16}$	$1.8*10^{-5}$	0.004
Height (cm)	175.7 ± 6.7	162.6 ± 6.2	$<2.2*10^{-16}$	176.0 ± 6.8	162.7 ± 6.2	$<2.2*10^{-16}$	$<2.2*10^{-16}$	$4.1*10^{-6}$
Weight (kg)	86.3 ± 14.5	71.8 ± 14.2	$<2.2*10^{-16}$	86.1 ± 14.2	71.4 ± 13.9	$<2.2*10^{-16}$	0.02	$2.2*10^{-8}$
BMI (kg/m2)	27.9 ± 4.3	27.2 ± 5.2	$<2.2*10^{-16}$	27.8 ± 4.2	27.0 ± 5.12	$<2.2*10^{-16}$	$5.9*10^{-12}$	$1.1*10^{-13}$
Waist circumference (cm)	97.4 ± 11.5	85.0 ± 12.7	$<2.2*10^{-16}$	96.9 ± 11.3	84.4 ± 12.4	$<2.2*10^{-16}$	$5.9*10^{-13}$	$<2.2*10^{-16}$
Hip circumference (cm)	103.6 ± 7.7	103.5 ± 7.5	0.73	103.5 ± 7.6	103.3 ± 10.2	$3.29*10^{-9}$	0.01	$6.4*10^{-7}$
Waist-to-hip ratio	0.94 ± 0.1	0.82 ± 0.1	$<2.2*10^{-16}$	0.93 ± 0.1	0.82 ± 0.1	$<2.2*10^{-16}$	$<2.2*10^{-16}$	$<2.2*10^{-16}$
Impedance measurements								
Total fat mass (kg)	22.6 ± 8.4	27.2 ± 10.1	$<2.2*10^{-16}$	22.3 ± 8.2	26.8 ± 10.0	$<2.2*10^{-16}$	$1.6*10^{-9}$	$2.7*10^{-11}$
Leg fat mass (kg)	6.3 ± 2.5	10.6 ± 3.6	$<2.2*10^{-16}$	6.2 ± 2.5	10.4 ± 3.5	$<2.2*10^{-16}$	$5.3*10^{-10}$	$8.7*10^{-13}$
Arm fat mass (kg)	2.2 ± 1.0	2.9 ± 1.6	$<2.2*10^{-16}$	2.2 ± 1.0	2.8 ± 1.5	$<2.2*10^{-16}$	$9.1*10^{-7}$	$1.6*10^{-12}$
Trunk fat mass (kg)	14.0 ± 5.1	13.8 ± 5.3	$<2.2*10^{-16}$	13.9 ± 5.0	13.6 ± 5.2	$<2.2*10^{-16}$	$3.4*10^{-8}$	$1.9*10^{-9}$
Proportional distribution of body fat								
Leg fat ratio – LFR (%)	28.0 ± 3.4	39.7 ± 4.0	$<2.2*10^{-16}$	28.1 ± 3.3	39.7 ± 4.1	$<2.2*10^{-16}$	0.02	0.19
Arm fat ratio – AFR (%)	9.9 ± 1.3	10.1 ± 1.6	$<2.2*10^{-16}$	9.9 ± 1.3	10.1 ± 1.6	$<2.2*10^{-16}$	0.03	$5.7*10^{-11}$
Trunk fat ratio – TFR (%)	62.2 ± 3.6	50.3 ± 4.1	$<2.2*10^{-16}$	62.2 ± 3.6	50.3 ± 4.1	$<2.2*10^{-16}$	0.22	0.19

Supplementary Table 2. SNP-heritability (*h2*) and LD score regression intercept estimates of body fat ratios. *h2* and LD score regression intercepts were generated by the LDSC method[1] (see online methods).

Females	***h2* (95%CI)**	**GC**	**LD score regression intercepts (± 95% CI)**
AFR females	0.25 ± 0.03	1.246	1.02 ± 0.02
LFR females	0.21 ± 0.03	1.207	1.03 ± 0.02
TFR females	0.23 ± 0.03	1.214	1.03 ± 0.02
Males			
AFR males	0.15 ± 0.02	1.146	1.01 ± 0.01
LFR males	0.13 ± 0.02	1.114	1.00 ± 0.01
TFR males	0.11 ± -0.02	1.105	1.00 ± 0.01

GC = genomic inflation factor (also denoted as λ_{GC}). h2 = heritability. CI = confidence interval.

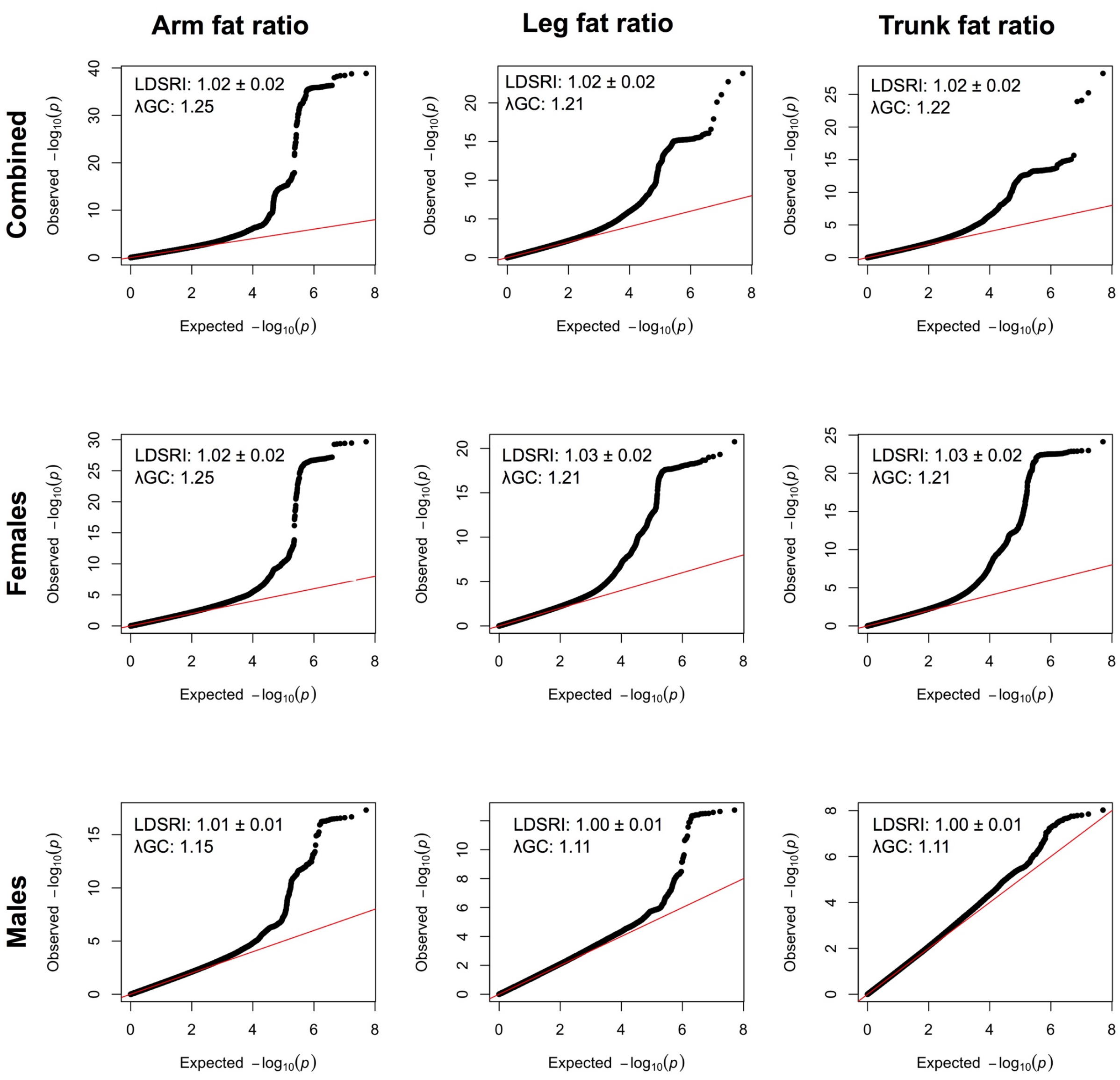

Supplementary Figure 1. Quantile-quantile plots of the distribution of p-values from GWAS of body fat ratios in the UK Biobank sex-combined, female and male discovery cohorts. LD-score regression intercepts (LDSRI) and genomic inflation factors were calculated using the LDSC software package. The red line shows the theoretical distribution of P-values under the null-hypothesis, i.e. there are no associations between genetic variants and the studied traits.

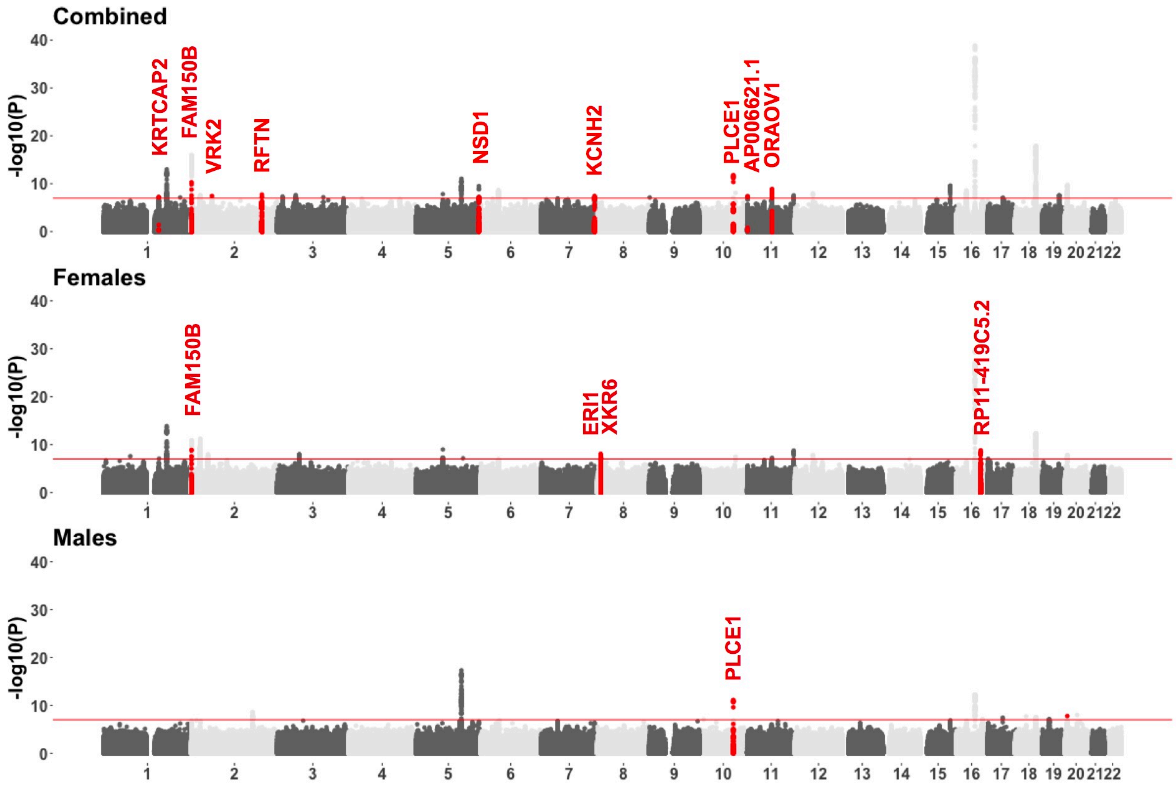

Supplementary Figure 2. Manhattan plots for GWAS results for arm fat ratio (AFR) in the combined discovery cohort and sex-stratified analyses. X-axes denote chromosome numbers. Y-axes denote the negative logarithm to base ten of P-values for association. Loci that have not been associated with an anthropometric trait in previous GWAS are highlighted in red.

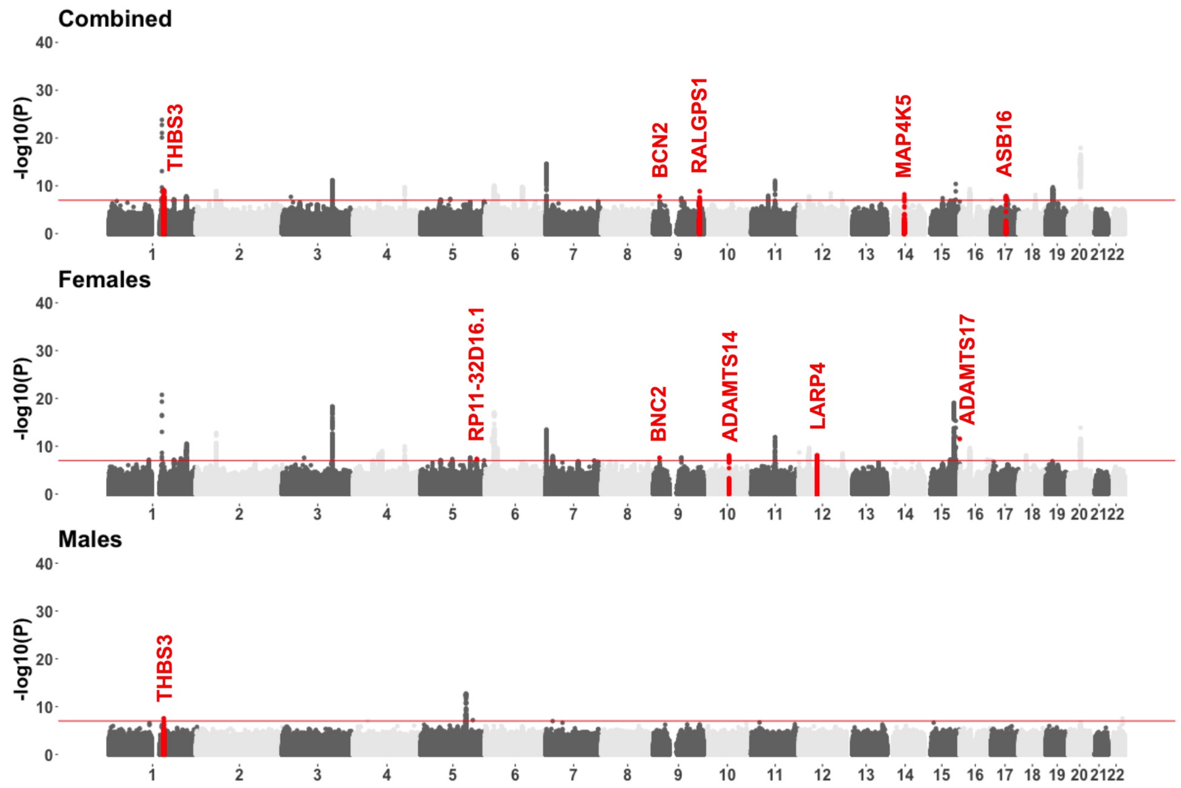

Supplementary Figure 3. Manhattan plots for GWAS results for leg fat ratio (LFR) in the combined discovery cohort and sex-stratified analyses. X-axes denote chromosome numbers. Y-axes denote the negative logarithm to base ten of P-values for association. Loci that have not been associated with an anthropometric trait in previous GWAS are highlighted in red.

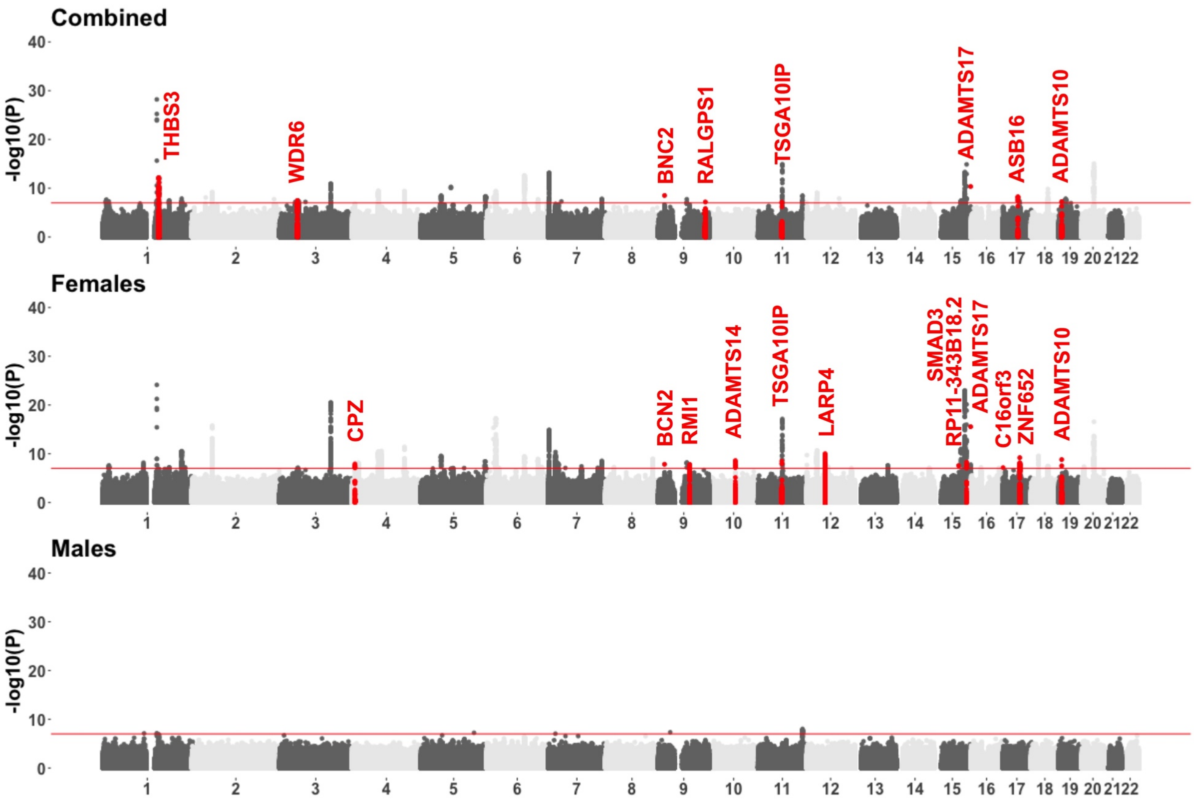

Supplementary Figure 4. Manhattan plots for GWAS results for trunk fat ratio (TFR) in the combined discovery cohort and sex-stratified analyses. X-axes denote chromosome numbers. Y-axes denote the negative logarithm to base ten of P-values for association. Loci that have not been associated with an anthropometric trait in previous GWAS are highlighted in red.

Supplementary Table 3. Independent signals* for association with body fat ratios in the combined cohort.

				Discovery						Replication					
**CHR**	**RSID**	**BP**	**Locus name**	β_{AFR}	P_{AFR}	β_{LFR}	P_{LFR}	β_{TFR}	P_{TFR}	β_{AFR}	P_{AFR}	β_{LFR}	P_{LFR}	β_{TFR}	P_{TFR}
1	rs4846204	10,308,958	*KIF1B (b)*	-0.008	1.94E-01	-0.032	5.41E-07	0.035	2.87E-08	-0.011	9.60E-03	-0.021	7.61E-07	0.026	2.25E-09
1	rs55750792	17,306,870	*MFAP2 (a)*	0.007	7.89E-02	0.020	1.90E-06	-0.023	6.94E-08	0.011	7.03E-05	0.013	1.15E-05	-0.018	6.19E-10
1	rs2273368	113,063,771	*WNT2B (a)*	0.009	9.81E-02	0.020	2.55E-04	-0.023	2.45E-05	0.006	8.89E-02	0.013	4.86E-04	-0.015	5.34E-05
1	rs11205303	149,906,413	*SF3B4 (a)*	-0.015	6.46E-04	-0.044	3.70E-24	0.048	1.74E-28	-0.005	9.80E-02	-0.052	3.24E-70	0.053	1.16E-72
1	rs4971091	155,143,768	*KRTCAP2 (b)*	-0.024	8.23E-08	0.006	2.05E-01	0.004	3.69E-01	-0.013	1.35E-05	0.005	8.48E-02	-0.001	8.58E-01
1	rs180921974	155,268,131	*THBS3(d)*	0.043	2.55E-03	0.087	1.18E-09	-0.102	1.05E-12	0.023	1.84E-02	0.090	1.47E-20	-0.097	9.45E-24
1	rs545608	177,899,121	*SEC16B (a)*	0.038	1.62E-13	-0.021	3.87E-05	0.007	2.06E-01	0.039	8.79E-28	-0.014	8.62E-05	-0.001	8.66E-01
1	rs5779197	184,009,826	*TSEN15, GLT25D2 (a)*	-0.004	4.31E-01	-0.024	8.60E-08	0.024	6.75E-08	-0.014	3.14E-06	-0.016	1.30E-07	0.019	1.52E-10
1	rs991967	218,615,451	*TGFB2 (a)*	-0.001	8.15E-01	-0.026	2.92E-08	0.026	4.76E-08	0.000	9.70E-01	-0.013	5.31E-05	0.012	8.43E-05
1	rs2820443	219,753,509	*LYPLAL1 (a)*	0.004	3.59E-01	-0.025	6.01E-08	0.022	3.16E-06	0.000	9.51E-01	-0.027	1.77E-17	0.025	4.20E-15
1	rs10916174	227,804,041	*ZNF678 (a)*	0.027	3.88E-06	0.012	3.93E-02	-0.022	1.49E-04	0.013	7.16E-04	0.013	1.04E-03	-0.017	1.90E-05
2	rs62107261	422,144	*FAM150B (a)*	-0.063	3.08E-10	0.019	0.05793	0.005	0.5976	-0.077	3.67E-30	0.033	8.53E-07	-0.0001	0.9784
2	rs6731872	624,205	*TMEM18 (a)*	-0.047	1.87E-16	0.006	3.16E-01	0.014	1.10E-02	-0.042	9.07E-29	0.006	1.12E-01	0.011	3.88E-03
2	rs754537	25,176,277	*RBJ, DNAJC27 (a)*	-0.021	7.03E-07	0.003	4.84E-01	0.006	1.78E-01	-0.024	1.00E-16	-0.003	2.96E-01	0.013	6.40E-06
2	rs17511102	37,960,613	*CDC42EP3 (a)*	-0.021	4.90E-03	-0.034	4.21E-06	0.042	1.10E-08	-0.006	2.45E-01	-0.019	1.86E-04	0.020	8.40E-05
2	rs7562173	46,976,217	*SOCS5 (a)*	-0.014	1.07E-03	-0.002	6.00E-01	0.010	2.92E-02	-0.009	3.66E-03	-0.007	2.43E-02	0.010	8.01E-04
2	rs3791679	56,096,892	*EFEMP1 (a)*	0.006	2.51E-01	0.030	1.86E-09	-0.031	8.73E-10	0.008	1.43E-02	0.036	3.25E-26	-0.039	1.24E-30
2	rs13011472	57,961,602	*VRK2 (b)*	-0.024	4.81E-08	0.007	1.30E-01	0.003	5.60E-01	-0.013	6.96E-06	0.001	8.04E-01	0.005	7.84E-02
2	rs148812496	198,540,352	*RFTN (b)*	-0.030	2.41E-08	-0.009	1.02E-01	0.019	3.67E-04	-0.015	4.19E-05	-0.012	9.29E-04	0.017	2.21E-06
3	rs4521268	49,137,904	*WDR6(c)*	0.007	1.44E-01	0.023	3.93E-07	-0.025	4.30E-08	0.006	6.27E-02	0.018	4.04E-09	-0.021	5.00E-12
3	rs1986599	50,034,637	*RBM6 (b)*	0.020	2.98E-03	0.028	2.95E-05	-0.037	4.28E-08	0.023	1.32E-07	0.012	8.65E-03	-0.021	1.47E-06
3	rs13069365	51,016,959	*DOCK3 (a)*	-0.035	3.09E-08	-0.011	7.20E-02	0.024	1.64E-04	-0.018	1.80E-05	-0.013	3.59E-03	0.019	9.94E-06
3	rs565817433	52,718,183	*NEK4(c)*	0.013	2.29E-03	0.017	4.57E-05	-0.023	6.83E-08	0.007	2.19E-02	0.011	2.39E-04	-0.013	5.92E-06
3	rs9853018	141,101,961	*ZBTB38 (a)*	0.002	5.77E-01	-0.029	1.42E-11	0.028	3.23E-11	-0.005	8.23E-02	-0.025	1.45E-18	0.027	9.63E-21
4	rs2241069	8,602,798	*CPZ (b)*	-0.010	1.71E-02	-0.016	2.37E-04	0.021	1.41E-06	-0.010	7.70E-04	-0.011	9.97E-05	0.015	3.85E-07

4	rs79334166	17,859,466	*FAM184B, C4orf30 (a)*	0.005	4.32E-01	0.023	2.51E-04	-0.025	8.54E-05	0.005	2.17E-01	0.017	5.53E-05	-0.019	9.83E-06
4	rs4694504	73,496,691	*ADAMTS3 (a, c)*	-0.015	4.13E-04	-0.018	1.94E-05	0.022	2.99E-07	-0.008	4.02E-03	-0.025	4.29E-18	0.028	5.98E-23
4	rs994014	82,165,790	*PRKG2 (a)*	-0.009	4.43E-02	-0.018	5.56E-05	0.021	6.71E-06	-0.012	8.92E-05	-0.019	9.33E-10	0.023	3.02E-13
4	rs7680661	145,565,116	*HHIP (a)*	0.007	1.92E-01	0.033	7.68E-09	-0.035	5.92E-10	0.013	6.89E-04	0.036	3.19E-21	-0.041	1.16E-26
5	rs465983	55,812,130	*MAP3K1 (a)*	-0.007	1.71E-01	-0.026	1.48E-07	0.029	6.75E-09	-0.007	2.70E-02	-0.021	2.04E-10	0.024	2.65E-12
5	rs34341	74,934,009	*ANKDD1B(c)*	-0.024	1.99E-08	0.006	1.58E-01	0.003	5.55E-01	-0.016	3.80E-08	0.005	9.67E-02	0.002	4.89E-01
5	rs115912456	82,815,158	*VCAN (a)*	0.038	4.15E-04	0.057	7.21E-08	-0.069	7.84E-11	0.018	1.26E-02	0.046	2.43E-10	-0.052	8.78E-13
5	rs3812049	127,418,850	*SLC12A2 (b)*	0.033	1.39E-11	-0.023	4.15E-06	0.010	3.72E-02	0.037	1.42E-28	-0.022	3.54E-11	0.008	1.09E-02
5	rs1317415	157,952,404	*RP11-32D16.1 (b)*	0.002	6.30E-01	-0.017	2.13E-04	0.016	4.03E-04	0.005	1.23E-01	-0.015	2.75E-06	0.012	8.24E-05
5	rs351855	176,520,243	*FGFR4 (a)*	0.029	4.51E-10	0.002	6.09E-01	-0.012	9.71E-03	0.016	3.05E-07	0.006	7.51E-02	-0.012	1.86E-04
5	rs34022431	176,677,563	*NSD1 (b)*	0.071	8.55E-08	0.021	1.13E-01	-0.048	3.46E-04	0.040	7.12E-06	0.029	1.51E-03	-0.042	3.02E-06
5	rs888762	178,547,313	*ADAMTS2 (b)*	-0.009	4.98E-02	-0.023	3.63E-07	0.026	6.32E-09	-0.004	2.26E-01	-0.018	8.90E-09	0.020	1.34E-10
6	rs41271299	19,839,415	*ID4 (a)*	-0.017	7.16E-02	-0.047	7.82E-07	0.055	9.83E-09	-0.020	1.87E-03	-0.041	3.58E-10	0.047	5.26E-13
6	rs9358913	26,239,404	*HIST1H2BE (a)*	0.002	6.90E-01	0.028	6.14E-09	-0.028	8.44E-09	0.012	4.39E-04	0.029	2.72E-19	-0.031	6.60E-22
6	rs9469762	34,236,430	*HMGA1 (a)*	0.016	2.39E-01	-0.053	8.75E-05	0.047	5.07E-04	0.016	6.26E-02	-0.022	1.34E-02	0.017	5.52E-02
6	rs2492863	34,603,646	*C6orf106 (b)*	0.012	5.38E-02	-0.035	1.58E-08	0.029	2.52E-06	0.018	8.46E-06	-0.025	2.62E-09	0.017	3.58E-05
6	rs2635727	50,820,940	*TFAP2 (a)*	-0.029	2.98E-09	0.007	1.39E-01	0.004	4.34E-01	-0.020	2.48E-09	0.012	2.09E-04	-0.004	2.44E-01
6	rs314263	105,392,745	*LIN28B (a)*	-0.012	6.92E-03	-0.029	2.46E-10	0.033	3.99E-13	-0.014	1.08E-05	-0.033	7.15E-27	0.036	1.87E-32
6	rs6570507	142,679,572	*GPR126 (a)*	0.012	1.06E-02	0.021	7.82E-06	-0.025	7.66E-08	0.009	4.95E-03	0.029	6.36E-20	-0.033	1.20E-25
6	rs2982708	152,356,220	*ESR1 (a)*	-0.015	2.25E-03	-0.010	3.05E-02	0.016	8.44E-04	-0.003	2.77E-01	-0.016	3.29E-07	0.017	6.45E-08
7	rs798491	2,800,521	*AMZ1 (a)*	-0.001	7.82E-01	0.036	5.20E-15	-0.034	1.16E-13	0.000	9.62E-01	0.034	7.71E-27	-0.033	1.11E-26
7	rs3823974	20,442,796	*ITGB8 (b)*	0.007	1.22E-01	0.019	1.86E-05	-0.021	1.38E-06	0.009	2.79E-03	0.015	2.88E-07	-0.018	1.85E-09
7	rs481806	28,207,300	*JAZF1 (a)*	-0.019	2.86E-05	-0.016	3.82E-04	0.024	2.90E-07	-0.016	5.70E-07	-0.020	1.04E-10	0.026	1.13E-16
7	rs527582137	92,251,321	*CDK6 (a)*	0.000	9.66E-01	-0.013	6.63E-03	0.013	6.61E-03	0.001	8.41E-01	-0.021	1.76E-10	0.021	4.75E-10
7	rs112416074	148,647,760	*PDIA4 (a)*	-0.007	2.05E-01	-0.029	2.85E-07	0.032	2.07E-08	-0.008	4.42E-02	-0.016	1.82E-05	0.019	9.60E-07
7	rs56282717	150,657,095	*KCNH2 (b)*	-0.027	5.00E-08	0.000	9.52E-01	0.010	4.81E-02	-0.020	5.59E-09	0.002	5.27E-01	0.006	9.83E-02
8	rs2044387	8,907,950	*ERI1 (b)*	0.018	4.00E-05	0.002	5.90E-01	-0.010	3.08E-02	0.017	3.06E-09	0.002	5.62E-01	-0.008	5.30E-03
8	rs12546366	10,802,146	*XKR6 (b)*	0.020	4.85E-06	0.004	3.13E-01	-0.011	8.68E-03	0.020	1.30E-11	0.007	2.33E-02	-0.013	3.25E-06

8	rs4733727	130,731,484	*MLZE, GSDMC (a)*	0.007	9.35E-02	0.018	4.07E-05	-0.020	2.81E-06	0.002	4.07E-01	0.011	8.31E-05	-0.012	2.27E-05
9	rs10962638	16,846,111	*BNC2 (b)*	0.005	4.50E-01	0.035	2.17E-08	-0.037	4.22E-09	0.000	9.97E-01	0.015	1.63E-04	-0.014	3.90E-04
9	rs35344761	78,510,823	*PCSK5 (a)*	0.003	6.46E-01	0.036	5.12E-08	-0.035	9.04E-08	0.014	1.57E-03	0.034	5.63E-14	-0.038	1.36E-17
9	rs7039458	86,639,999	*RMI1 (c,d)*	0.001	8.97E-01	-0.024	6.78E-07	0.025	5.61E-07	-0.003	3.27E-01	-0.024	1.57E-12	0.024	1.79E-13
9	rs3780327	129,945,847	*RALGPS1 (b)*	0.008	1.36E-01	-0.031	1.95E-09	0.028	8.70E-08	0.010	5.98E-03	-0.016	2.50E-06	0.013	2.06E-04
10	rs34821335	72,433,203	*ADAMTS14(c)*	-0.010	3.91E-02	-0.017	5.50E-04	0.020	2.48E-05	-0.008	1.42E-02	-0.014	1.20E-05	0.016	7.67E-07
10	rs11289753	96,026,184	*PLCE1 (b)*	0.030	2.41E-12	0.007	1.24E-01	-0.017	6.15E-05	0.021	1.60E-12	0.007	2.21E-02	-0.013	4.68E-06
11	rs1138714	825,110	*AP006621.1 (b)*	-0.024	5.47E-08	0.002	5.93E-01	0.007	1.21E-01	-0.012	2.50E-05	0.002	6.05E-01	0.004	1.88E-01
11	rs10769282	47,626,492	*MTCH2 (a)*	-0.022	1.50E-06	0.026	1.49E-08	-0.017	2.20E-04	-0.018	4.99E-09	0.021	8.93E-12	-0.014	6.53E-06
11	rs71455793	65,715,204	*TSGA10IP(d)*	0.033	1.21E-03	0.047	3.28E-06	-0.054	9.09E-08	0.017	1.12E-02	0.015	2.63E-02	-0.019	5.96E-03
11	rs552846225	66,913,469	*KDM2A (a)*	0.026	3.82E-04	0.049	1.26E-11	-0.057	3.32E-15	0.014	6.06E-03	0.034	3.33E-12	-0.039	3.94E-15
11	rs4980661	69,306,579	*MYEOV, CCND1 (a)*	0.024	9.49E-09	-0.008	5.64E-02	-0.002	5.72E-01	0.013	9.61E-06	0.003	3.80E-01	-0.007	1.33E-02
11	rs1789166	69,482,091	*ORAOV1 (b)*	-0.027	1.85E-09	0.010	1.89E-02	0.000	9.13E-01	-0.013	2.05E-05	0.002	4.08E-01	0.002	5.21E-01
12	rs34716573	576,037	*B4GALNT3 (a)*	-0.003	4.79E-01	-0.022	1.02E-06	0.024	1.22E-07	-0.001	6.53E-01	-0.009	2.16E-03	0.009	3.97E-03
12	rs11049566	28,532,961	*CCDC91 (a)*	0.012	1.09E-02	0.026	2.46E-08	-0.028	1.36E-09	0.015	3.84E-06	0.013	2.84E-05	-0.018	6.50E-09
12	rs7306275	50,250,111	*BCDIN3D, FAIM2 (a)*	0.025	1.36E-08	-0.018	4.73E-05	0.008	7.74E-02	0.016	4.15E-08	-0.010	8.11E-04	0.003	3.55E-01
12	rs11614785	50,880,422	*LARP4 (b)*	-0.014	1.36E-03	-0.014	1.40E-03	0.019	1.77E-05	-0.012	8.80E-05	-0.009	3.43E-03	0.013	1.76E-05
12	rs6489111	123,051,018	*KNTC1 (a)*	0.007	1.49E-01	-0.023	7.51E-07	0.019	3.71E-05	0.010	6.24E-04	-0.011	3.89E-04	0.006	3.96E-02
13	rs6492538	91,993,746	*GPC5 (b)*	-0.005	2.99E-01	-0.020	1.38E-04	0.020	9.86E-05	-0.004	2.11E-01	-0.009	1.07E-02	0.010	3.79E-03
14	rs71420186	50,960,918	*MAP4K5 (b)*	0.029	7.94E-04	-0.050	8.25E-09	0.036	2.55E-05	0.011	5.94E-02	-0.038	3.20E-11	0.033	1.17E-08
15	rs35874463	67,457,698	*SMAD3(d)*	-0.020	2.30E-02	-0.030	8.59E-04	0.039	1.86E-05	-0.009	1.31E-01	-0.046	4.96E-14	0.047	1.61E-14
15	rs12905253	74,232,437	*PML (a)*	0.016	1.75E-04	0.018	1.34E-05	-0.023	4.43E-08	0.012	5.03E-05	0.018	1.60E-10	-0.022	6.32E-15
15	rs11856122	84,576,348	*ADAMTSL3 (a)*	0.026	6.59E-10	0.021	5.01E-07	-0.031	3.68E-13	0.028	4.40E-23	0.025	1.65E-18	-0.034	1.07E-32
15	rs11856122	89,361,669	*RP11-343B18.2(c)*	-0.007	1.07E-01	-0.019	7.64E-06	0.020	1.39E-06	-0.006	5.31E-02	-0.014	2.53E-06	0.015	1.00E-07
15	rs28584580	89,397,827	*ACAN (a)*	0.040	1.43E-03	0.029	2.12E-02	-0.043	6.58E-04	0.043	3.15E-07	0.044	1.97E-07	-0.058	7.09E-12
15	rs3817428	89,415,247	*ACAN (a)*	0.021	1.53E-05	0.031	6.01E-11	-0.038	2.30E-15	0.024	2.32E-13	0.026	4.34E-16	-0.034	4.59E-25
15	rs72755233	100,692,953	*ADAMTS17(d)*	0.030	6.56E-06	0.034	2.53E-07	-0.044	6.43E-11	0.033	1.42E-13	0.042	3.25E-20	-0.053	1.54E-31

15	rs4988781	100,800,840	*ADAMTS17 (a)*	-0.008	8.21E-02	-0.015	1.17E-03	0.018	5.28E-05	-0.006	3.58E-02	-0.019	1.07E-09	0.021	6.50E-12
16	rs369515117	28,862,780	*SH2B1 (a)*	0.014	1.81E-03	-0.027	1.99E-09	0.021	2.47E-06	0.019	1.82E-10	-0.025	1.92E-16	0.016	1.23E-07
16	rs8050894	31,104,509	*KAT8(c)*	-0.024	7.02E-08	0.002	6.14E-01	0.005	2.18E-01	-0.017	4.76E-09	0.010	1.00E-03	-0.003	2.86E-01
16	rs55872725	53,809,123	*FTO(a)*	0.056	6.11E-39	-0.006	1.85E-01	-0.018	2.28E-05	0.050	4.99E-66	-0.009	3.22E-03	-0.013	7.17E-06
16	rs8057620	69,884,619	*RP11-419C5.2(c)*	0.015	4.71E-04	-0.013	3.19E-03	0.005	2.19E-01	0.017	1.49E-09	-0.010	4.30E-04	0.003	3.25E-01
16	rs10584116	90,062,323	*C16orf3(c)*	0.010	1.66E-01	0.027	1.47E-04	-0.030	2.62E-05	0.011	2.42E-02	0.015	1.69E-03	-0.018	1.67E-04
17	rs2071167	42,287,519	*ASB16(c)*	-0.005	2.79E-01	-0.028	1.84E-08	0.029	7.59E-09	0.004	1.94E-01	-0.013	1.04E-04	0.011	9.54E-04
17	rs2074188	45,888,251	*SP6, SP2 (a)*	-0.001	7.67E-01	-0.023	9.65E-08	0.023	4.73E-08	-0.006	2.69E-02	-0.009	2.85E-03	0.011	1.83E-04
17	rs28394864	47,450,775	*ZNF652 (b)*	0.011	1.31E-02	0.018	1.98E-05	-0.022	1.42E-07	0.016	7.48E-08	0.014	1.91E-06	-0.018	3.25E-10
18	rs4800148	20,724,328	*RBBP8, CABLES1, C18orf45 (a)*	0.007	1.51E-01	0.016	1.80E-03	-0.018	5.30E-04	0.011	2.14E-03	0.015	1.70E-05	-0.019	8.98E-08
18	rs7236575	46,653,380	*DYM (a)*	0.011	7.46E-02	0.035	1.07E-08	-0.039	2.14E-10	0.007	1.05E-01	0.011	6.36E-03	-0.014	7.66E-04
18	rs71336392	57,850,927	*MC4R (a)*	0.044	2.46E-18	-0.011	2.70E-02	-0.008	1.23E-01	0.048	3.63E-46	-0.012	2.40E-04	-0.006	6.88E-02
19	rs62621197	8,670,147	*ADAMTS10(d)*	0.032	1.14E-02	0.059	4.78E-06	-0.069	7.38E-08	0.036	1.35E-05	0.085	8.32E-25	-0.097	2.43E-31
19	rs10402308	19,657,500	*GATAD2A (a)*	-0.008	1.73E-01	0.035	2.82E-10	-0.031	2.03E-08	0.000	9.62E-01	0.014	1.33E-04	-0.015	8.56E-05
19	rs12971970	47,559,273	*ZC3H4 (a)*	-0.028	3.29E-08	0.004	4.32E-01	0.006	2.03E-01	-0.018	4.81E-08	0.001	7.97E-01	0.006	8.69E-02
20	rs6085551	6,408,920	*RP11-199O14.1 (b)*	0.020	2.10E-06	0.005	2.81E-01	-0.010	1.53E-02	0.015	2.95E-07	0.007	1.01E-02	-0.012	5.96E-05
20	rs2145270	6,621,685	*BMP2 (a)*	-0.028	2.53E-10	-0.005	2.48E-01	0.014	8.84E-04	-0.021	2.69E-12	-0.007	2.37E-02	0.014	2.31E-06
20	rs143384	34,025,756	*GDF5 (a)*	0.010	2.34E-02	-0.0376	2.38E-18	0.034	1.62E-15	0.005	1.19E-01	-0.031	7.44E-27	0.030	3.38E-24

* - The 'clump' function in PLINK was used to identify signals for association that were independent in each GWAS (see methods). Several of the regions were associated with more than one of the three body fat ratios or associated with one body fat ratio in more than one of the strata (males, females or sex-combined). As a result, different leading SNPs were observed for different traits and strata at several loci. To assess whether these represented the same signal, we assessed the LD between the leading SNPs for different traits. SNPs in low LD with the most strongly associated SNP at each locus (R2-value < 0.05) were considered to represent independent signals. The 'Locus' column denotes: (a) the reported gene from previous GWAS, (b) the most proximal gene, (c) the most strongly associated gene that is regulated by an eQTL in LD with the leading SNP, or (d) a gene containing a missense variant in LD with the leading SNP. Direction - summary of effect direction for the discovery and replication cohorts, with one '+' or '-' per cohort. All gene names are in italic font. Further data as well as annotations are available in Supplementary Data 1.

Supplementary Table 4. Independent signals* for association with body fat ratios in females.

				Discovery						Replication					
**CHR**	**RSID**	**BP**	**Locus name**	β_{AFR}	P_{AFR}	β_{LFR}	P_{LFR}	β_{TFR}	P_{TFR}	β_{AFR}	P_{AFR}	β_{LFR}	P_{LFR}	β_{TFR}	P_{TFR}
1	rs4846204	10,308,958	*KIF1B (b)*	-0.011	2.20E-01	-0.035	6.60E-05	0.039	6.77E-06	-0.010	1.01E-01	-0.019	9.38E-04	0.025	2.29E-05
1	rs55750792	17,306,870	*MFAP2 (a)*	0.016	6.48E-03	0.027	4.76E-06	-0.032	3.21E-08	0.019	1.36E-06	0.019	8.15E-07	-0.027	3.04E-12
1	rs2273368	113,063,771	*WNT2B (a)*	0.009	2.49E-01	0.040	8.70E-08	-0.043	9.44E-09	0.010	3.80E-02	0.030	9.29E-10	-0.033	1.09E-11
1	rs11205303	149,906,413	*SF3B4 (a)*	-0.016	7.23E-03	-0.056	3.89E-21	0.060	1.82E-24	-0.012	2.51E-03	-0.063	1.67E-57	0.067	5.98E-65
1	rs4971091	155,143,768	*KRTCAP2 (b)*	-0.030	7.74E-07	0.003	5.93E-01	0.009	1.21E-01	-0.013	1.27E-03	0.007	9.42E-02	-0.001	7.78E-01
1	rs180921974	155,268,131	*THBS3(d)*	0.050	1.06E-02	0.082	2.65E-05	-0.101	2.73E-07	0.027	4.08E-02	0.095	4.45E-13	-0.103	3.82E-15
1	rs545608	177,899,121	*SEC16B (a)*	0.054	4.02E-14	-0.029	6.82E-05	0.008	2.54E-01	0.048	5.37E-24	-0.027	2.10E-08	0.008	9.20E-02
1	rs5779197	184,009,826	*TSEN15, GLT25D2 (a)*	-0.004	5.19E-01	-0.032	2.91E-07	0.032	3.34E-07	-0.018	1.41E-05	-0.031	6.80E-14	0.035	2.32E-17
1	rs991967	218,615,451	*TGFB2 (a)*	-0.011	8.11E-02	-0.039	1.07E-09	0.043	4.34E-11	-0.002	7.03E-01	-0.024	3.62E-08	0.024	1.82E-08
1	rs2820443	219,753,509	*LYPLAL1 (a)*	0.005	4.00E-01	-0.042	5.55E-11	0.037	6.95E-09	0.001	8.28E-01	-0.040	6.34E-21	0.037	5.95E-18
1	rs10916174	227,804,041	*ZNF678 (a)*	0.038	2.81E-06	0.028	4.96E-04	-0.043	8.05E-08	0.021	7.29E-05	0.030	1.86E-08	-0.037	7.54E-12
2	rs62107261	422,144	*FAM150B (a)*	-0.080	5.17E-09	0.032	2.05E-02	-0.002	8.82E-01	-0.094	1.92E-25	0.047	2.08E-07	-0.006	5.35E-01
2	rs6731872	624,205	*TMEM18 (a)*	-0.052	1.92E-11	0.021	5.97E-03	0.003	6.66E-01	-0.051	5.88E-23	0.018	4.19E-04	0.004	4.20E-01
2	rs754537	25,176,277	*RBJ, DNAJC27 (a)*	-0.040	9.90E-12	0.004	4.57E-01	0.011	5.01E-02	-0.044	2.00E-29	-0.006	1.34E-01	0.024	1.37E-09
2	rs17511102	37,960,613	*CDC42EP3 (a)*	-0.037	3.03E-04	-0.030	2.92E-03	0.044	1.30E-05	-0.018	8.82E-03	-0.023	6.31E-04	0.028	2.92E-05
2	rs7562173	46,976,217	*SOCS5 (a)*	-0.034	1.35E-08	-0.005	3.68E-01	0.020	1.02E-03	-0.017	1.20E-05	-0.008	4.38E-02	0.014	3.85E-04
2	rs3791679	56,096,892	*EFEMP1 (a)*	0.020	3.54E-03	0.051	3.60E-13	-0.057	3.14E-16	0.022	2.29E-06	0.052	5.06E-29	-0.060	2.66E-38
2	rs13011472	57,961,602	*VRK2 (b)*	-0.025	1.85E-05	0.003	6.60E-01	0.007	2.06E-01	-0.014	2.49E-04	0.000	9.48E-01	0.006	1.18E-01
2	rs148812496	198,540,352	*RFTN (b)*	-0.031	3.63E-05	0.000	9.89E-01	0.011	1.52E-01	-0.017	5.18E-04	-0.012	1.10E-02	0.018	2.58E-04
3	rs4521268	49,137,904	*WDR6(c)*	0.012	5.85E-02	0.028	6.81E-06	-0.033	1.19E-07	0.011	1.05E-02	0.019	2.58E-06	-0.025	2.57E-09
3	rs1986599	50,034,637	*RBM6 (b)*	0.036	1.05E-04	0.027	3.90E-03	-0.043	5.54E-06	0.027	6.78E-06	0.007	2.33E-01	-0.018	2.28E-03
3	rs13069365	51,016,959	*DOCK3 (a)*	-0.030	6.35E-04	-0.024	5.76E-03	0.034	8.06E-05	-0.021	3.77E-04	-0.017	3.67E-03	0.023	5.73E-05
3	rs565817433	52,718,183	*NEK4(c)*	0.020	5.40E-04	0.015	9.74E-03	-0.023	1.05E-04	0.008	4.65E-02	0.017	1.51E-05	-0.021	1.30E-07
3	rs9853018	141,101,961	*ZBTB38 (a)*	-0.008	1.87E-01	-0.052	9.42E-19	0.055	1.12E-20	-0.022	1.38E-08	-0.047	1.34E-33	0.055	4.84E-45

4	rs2241069	8,602,798	*CPZ (b)*	-0.014	1.92E-02	-0.026	6.68E-06	0.033	1.56E-08	-0.017	1.22E-05	-0.013	1.38E-03	0.019	7.86E-07
4	rs79334166	17,859,466	*FAM184B, C4orf30 (a)*	0.029	8.05E-04	0.040	5.23E-06	-0.050	1.04E-08	0.035	1.78E-09	0.015	1.13E-02	-0.027	2.96E-06
4	rs4694504	73,496,691	*ADAMTS3 (a, c)*	-0.018	1.66E-03	-0.029	7.98E-07	0.033	1.62E-08	-0.012	1.59E-03	-0.032	8.82E-17	0.038	4.69E-22
4	rs994014	82,165,790	*PRKG2 (a)*	-0.014	2.66E-02	-0.038	1.42E-09	0.042	4.03E-11	-0.014	9.20E-04	-0.030	8.26E-13	0.036	3.70E-17
4	rs7680661	145,565,116	*HHIP (a)*	0.015	4.95E-02	0.047	1.40E-09	-0.052	2.04E-11	0.023	6.80E-06	0.058	6.44E-29	-0.067	3.57E-38
5	rs465983	55,812,130	*MAP3K1 (a)*	-0.020	2.84E-03	-0.036	1.82E-07	0.043	3.88E-10	-0.013	3.95E-03	-0.025	2.71E-08	0.029	1.92E-10
5	rs34341	74,934,009	*ANKDD1B(c)*	-0.036	1.47E-09	0.013	3.36E-02	0.001	8.26E-01	-0.025	1.20E-10	0.016	5.66E-05	-0.006	1.56E-01
5	rs115912456	82,815,158	*VCAN (a)*	0.010	5.11E-01	0.072	9.77E-07	-0.077	1.51E-07	0.012	2.08E-01	0.072	1.47E-13	-0.076	9.65E-15
5	rs3812049	127,418,850	*SLC12A2 (b)*	0.009	1.66E-01	0.001	8.49E-01	-0.004	5.16E-01	0.021	1.99E-06	-0.011	1.07E-02	0.004	4.16E-01
5	rs1317415	157,952,404	*RP11-32D16.1 (b)*	0.011	8.66E-02	-0.034	6.11E-08	0.030	1.53E-06	0.004	3.45E-01	-0.018	1.81E-05	0.015	3.49E-04
5	rs351855	176,520,243	*FGFR4 (a)*	0.024	1.58E-04	0.015	1.67E-02	-0.023	2.57E-04	0.012	4.43E-03	0.012	6.50E-03	-0.017	8.57E-05
5	rs34022431	176,677,563	*NSD1 (b)*	0.081	7.79E-06	0.041	2.40E-02	-0.074	4.83E-05	0.057	2.51E-06	0.040	1.02E-03	-0.060	7.68E-07
5	rs888762	178,547,313	*ADAMTS2 (b)*	-0.008	2.30E-01	-0.033	1.21E-07	0.037	5.48E-09	-0.007	8.66E-02	-0.023	1.79E-08	0.027	1.11E-10
6	rs41271299	19,839,415	*ID4 (a)*	-0.042	1.65E-03	-0.092	3.93E-12	0.107	8.09E-16	-0.039	9.65E-06	-0.077	1.74E-18	0.090	1.13E-24
6	rs9358913	26,239,404	*HIST1H2BE (a)*	0.008	2.39E-01	0.055	7.41E-17	-0.057	1.03E-17	0.010	2.98E-02	0.052	2.59E-31	-0.053	1.11E-32
6	rs9469762	34,236,430	*HMGA1 (a)*	0.014	4.51E-01	-0.101	8.01E-08	0.096	3.86E-07	0.011	3.37E-01	-0.059	7.42E-07	0.054	4.68E-06
6	rs2492863	34,603,646	*C6orf106 (b)*	0.017	4.82E-02	-0.053	3.48E-10	0.045	1.04E-07	0.022	7.87E-05	-0.046	5.26E-16	0.036	1.48E-10
6	rs2635727	50,820,940	*TFAP2 (a)*	-0.032	3.48E-06	0.009	1.77E-01	0.004	5.77E-01	-0.021	3.65E-06	0.012	7.24E-03	-0.004	3.81E-01
6	rs314263	105,392,745	*LIN28B (a)*	-0.019	1.95E-03	-0.032	2.93E-07	0.039	4.96E-10	-0.015	3.43E-04	-0.035	5.35E-17	0.039	8.42E-21
6	rs6570507	142,679,572	*GPR126 (a)*	0.027	3.41E-05	0.029	9.03E-06	-0.039	1.78E-09	0.013	2.42E-03	0.039	1.02E-19	-0.045	1.57E-25
6	rs2982708	152,356,220	*ESR1 (a)*	-0.023	5.23E-04	-0.028	1.85E-05	0.035	6.83E-08	-0.008	8.21E-02	-0.023	1.89E-07	0.025	1.35E-08
7	rs798491	2,800,521	*AMZ1 (a)*	0.009	1.66E-01	0.048	7.78E-14	-0.050	2.19E-15	0.012	6.34E-03	0.044	6.08E-25	-0.048	1.69E-29
7	rs3823974	20,442,796	*ITGB8 (b)*	0.016	8.65E-03	0.034	1.49E-08	-0.039	6.56E-11	0.011	5.57E-03	0.024	2.76E-09	-0.028	2.43E-12
7	rs481806	28,207,300	*JAZF1 (a)*	-0.020	1.69E-03	-0.028	9.99E-06	0.036	1.43E-08	-0.025	2.97E-09	-0.030	4.46E-12	0.039	2.31E-20
7	rs527582137	92,251,321	*CDK6 (a)*	-0.008	2.53E-01	-0.034	3.69E-07	0.037	4.98E-08	-0.007	9.83E-02	-0.030	2.67E-11	0.032	1.02E-12
7	rs112416074	148,647,760	*PDIA4 (a)*	-0.016	3.98E-02	-0.038	1.08E-06	0.046	3.88E-09	-0.019	3.75E-04	-0.023	7.98E-06	0.029	2.37E-08
7	rs56282717	150,657,095	*KCNH2 (b)*	-0.023	6.60E-04	-0.003	6.18E-01	0.012	7.62E-02	-0.021	2.65E-06	0.006	2.21E-01	0.003	5.18E-01

8	rs2044387	8,907,950	*ERI1 (b)*	0.035	1.14E-08	0.005	4.36E-01	-0.018	3.31E-03	0.024	1.03E-09	0.005	2.31E-01	-0.014	7.04E-04
8	rs12546366	10,802,146	*XKR6 (b)*	0.031	9.29E-08	0.004	4.97E-01	-0.017	4.60E-03	0.021	6.50E-08	0.004	2.81E-01	-0.012	1.85E-03
8	rs4733727	130,731,484	*MLZE, GSDMC (a)*	0.019	1.05E-03	0.029	1.04E-06	-0.035	1.66E-09	0.010	1.13E-02	0.015	1.03E-04	-0.020	4.82E-07
9	rs10962638	16,846,111	*BNC2 (b)*	0.001	8.66E-01	0.047	3.70E-08	-0.048	1.90E-08	0.000	9.51E-01	0.021	1.21E-04	-0.020	3.31E-04
9	rs35344761	78,510,823	*PCSK5 (a)*	0.008	3.79E-01	0.050	3.07E-08	-0.052	9.95E-09	0.021	4.43E-04	0.056	2.05E-20	-0.062	1.62E-24
9	rs7039458	86,639,999	*RMI1 (c,d)*	-0.016	1.95E-02	-0.032	2.04E-06	0.038	2.07E-08	-0.008	6.43E-02	-0.025	1.75E-08	0.028	3.32E-10
9	rs3780327	129,945,847	*RALGPS1 (b)*	0.012	1.01E-01	-0.028	9.94E-05	0.024	7.88E-04	0.013	4.59E-03	-0.012	1.23E-02	0.007	1.34E-01
10	rs34821335	72,433,203	*ADAMTS14(c)*	-0.002	7.37E-01	-0.038	1.08E-08	0.039	3.40E-09	-0.004	3.41E-01	-0.023	2.87E-07	0.023	2.22E-07
10	rs11289753	96,026,184	*PLCE1 (b)*	0.019	1.64E-03	0.016	6.86E-03	-0.024	4.25E-05	0.010	1.29E-02	0.017	1.12E-05	-0.020	6.16E-07
11	rs1138714	825,110	*AP006621.1 (b)*	-0.025	5.00E-05	0.001	8.24E-01	0.010	1.18E-01	-0.014	4.36E-04	0.002	6.83E-01	0.004	2.66E-01
11	rs10769282	47,626,492	*MTCH2 (a)*	-0.026	5.28E-05	0.028	1.57E-05	-0.018	6.08E-03	-0.021	8.94E-07	0.024	2.87E-08	-0.016	2.27E-04
11	rs71455793	65,715,204	*TSGA10IP(d)*	0.043	2.19E-03	0.069	6.22E-07	-0.082	4.45E-09	0.016	7.24E-02	0.035	1.45E-04	-0.039	1.85E-05
11	rs552846225	66,913,469	*KDM2A (a)*	0.043	1.37E-05	0.064	1.07E-10	-0.080	1.12E-15	0.023	4.32E-04	0.055	1.41E-16	-0.064	6.82E-22
11	rs4980661	69,306,579	*MYEOV, CCND1 (a)*	0.027	3.60E-06	-0.006	2.81E-01	-0.007	2.27E-01	0.012	1.78E-03	0.006	1.44E-01	-0.010	1.01E-02
11	rs1789166	69,482,091	*ORAOV1 (b)*	-0.033	1.14E-07	0.009	1.53E-01	0.005	4.48E-01	-0.014	4.97E-04	0.000	9.07E-01	0.006	1.64E-01
12	rs34716573	576,037	*B4GALNT3 (a)*	-0.007	2.59E-01	-0.037	2.82E-09	0.039	2.75E-10	-0.006	1.48E-01	-0.016	7.69E-05	0.017	2.56E-05
12	rs11049566	28,532,961	*CCDC91 (a)*	0.010	1.05E-01	0.040	4.93E-10	-0.042	6.78E-11	0.019	7.70E-06	0.022	4.27E-07	-0.028	4.07E-11
12	rs7306275	50,250,111	*BCDIN3D, FAIM2 (a)*	0.033	3.36E-08	-0.023	1.74E-04	0.009	1.52E-01	0.019	3.03E-06	-0.015	2.58E-04	0.006	1.31E-01
12	rs11614785	50,880,422	*LARP4 (b)*	-0.015	1.62E-02	-0.035	1.18E-08	0.040	1.37E-10	-0.015	4.86E-04	-0.020	1.05E-06	0.026	2.93E-10
12	rs6489111	123,051,018	*KNTC1 (a)*	0.011	7.41E-02	-0.037	4.48E-09	0.031	7.98E-07	0.007	7.63E-02	-0.019	4.52E-06	0.015	2.10E-04
13	rs6492538	91,993,746	*GPC5 (b)*	-0.011	1.21E-01	-0.036	3.32E-07	0.039	3.16E-08	-0.006	2.38E-01	-0.019	5.36E-05	0.021	8.33E-06
14	rs71420186	50,960,918	*MAP4K5 (b)*	0.024	4.19E-02	-0.044	1.84E-04	0.031	8.78E-03	0.009	2.41E-01	-0.033	2.79E-05	0.028	3.11E-04
15	rs35874463	67,457,698	*SMAD3(d)*	-0.042	7.15E-04	-0.053	2.04E-05	0.069	3.58E-08	-0.023	5.32E-03	-0.057	1.02E-11	0.064	1.44E-14
15	rs12905253	74,232,437	*PML (a)*	0.024	3.30E-05	0.031	8.42E-08	-0.039	1.58E-11	0.012	2.66E-03	0.027	2.38E-12	-0.032	1.65E-16
15	rs11856122	84,576,348	*ADAMTSL3 (a)*	0.022	1.94E-04	0.051	1.80E-18	-0.058	2.50E-23	0.030	1.08E-14	0.048	2.33E-35	-0.058	1.02E-49
15	rs11856122	89,361,669	*RP11-343B18.2(c)*	-0.013	2.74E-02	-0.030	3.02E-07	0.034	7.24E-09	-0.014	4.10E-04	-0.015	8.18E-05	0.020	2.71E-07

15	rs28584580	89,397,827	*ACAN (a)*	0.069	5.58E-05	0.091	8.51E-08	-0.113	3.98E-11	0.065	1.78E-08	0.096	8.69E-17	-0.118	6.82E-25
15	rs3817428	89,415,247	*ACAN (a)*	0.027	3.84E-05	0.053	8.29E-16	-0.061	1.59E-20	0.022	5.49E-07	0.045	2.43E-24	-0.051	3.78E-31
15	rs72755233	100,692,953	*ADAMTS17(d)*	0.038	3.87E-05	0.064	4.36E-12	-0.074	5.09E-16	0.046	4.81E-14	0.065	6.94E-26	-0.080	9.32E-39
15	rs4988781	100,800,840	*ADAMTS17 (a)*	-0.016	1.03E-02	-0.028	9.99E-06	0.035	2.22E-08	-0.015	2.28E-04	-0.025	1.86E-09	0.031	5.25E-14
16	rs369515117	28,862,780	*SH2B1 (a)*	0.015	1.45E-02	-0.039	3.31E-10	0.033	1.21E-07	0.023	4.52E-08	-0.030	3.32E-13	0.019	3.99E-06
16	rs8050894	31,104,509	*KAT8(c)*	-0.022	2.31E-04	0.003	6.48E-01	0.005	3.86E-01	-0.027	8.83E-12	0.016	1.07E-04	-0.005	2.42E-01
16	rs55872725	53,809,123	*FTO(a)*	0.067	6.75E-30	-0.009	1.16E-01	-0.020	6.28E-04	0.062	3.20E-55	-0.020	3.89E-07	-0.006	1.22E-01
16	rs8057620	69,884,619	*RP11-419C5.2(c)*	0.035	2.27E-09	-0.004	5.32E-01	-0.010	8.99E-02	0.024	4.40E-10	-0.007	6.13E-02	-0.003	4.06E-01
16	rs10584116	90,062,323	*C16orf3(c)*	0.011	2.81E-01	0.050	3.27E-07	-0.052	8.99E-08	0.008	1.95E-01	0.029	7.41E-06	-0.033	6.97E-07
17	rs2071167	42,287,519	*ASB16(c)*	0.003	6.40E-01	-0.031	5.91E-06	0.029	2.45E-05	0.011	1.75E-02	-0.023	1.05E-06	0.018	1.00E-04
17	rs2074188	45,888,251	*SP6, SP2 (a)*	-0.002	6.91E-01	-0.026	1.05E-05	0.027	2.98E-06	-0.007	8.82E-02	-0.017	1.46E-05	0.019	1.65E-06
17	rs28394864	47,450,775	*ZNF652 (b)*	0.016	6.06E-03	0.029	9.48E-07	-0.036	8.89E-10	0.013	6.51E-04	0.019	9.76E-07	-0.024	1.70E-09
18	rs4800148	20,724,328	*RBBP8, CABLES1, C18orf45 (a)*	0.017	1.81E-02	0.040	1.12E-08	-0.044	3.88E-10	0.013	5.30E-03	0.035	1.20E-13	-0.039	8.29E-17
18	rs7236575	46,653,380	*DYM (a)*	0.024	4.48E-03	0.038	5.64E-06	-0.046	4.65E-08	0.014	1.38E-02	0.017	2.84E-03	-0.022	1.33E-04
18	rs71336392	57,850,927	*MC4R (a)*	0.049	8.94E-13	-0.022	1.55E-03	0.001	9.17E-01	0.048	2.58E-26	-0.020	8.52E-06	0.000	9.94E-01
19	rs62621197	8,670,147	*ADAMTS10(d)*	0.055	1.81E-03	0.087	8.48E-07	-0.105	2.17E-09	0.060	9.82E-08	0.126	2.81E-29	-0.147	3.45E-39
19	rs10402308	19,657,500	*GATAD2A (a)*	0.010	1.84E-01	0.033	1.36E-05	-0.035	4.11E-06	0.013	9.38E-03	0.012	1.97E-02	-0.016	1.44E-03
19	rs12971970	47,559,273	*ZC3H4 (a)*	-0.029	2.38E-05	0.003	6.60E-01	0.008	2.38E-01	-0.021	5.17E-06	0.011	1.32E-02	-0.004	3.95E-01
20	rs6085551	6,408,920	*RP11-199O14.1 (b)*	0.007	2.27E-01	0.012	3.62E-02	-0.015	1.26E-02	0.018	4.71E-06	0.015	1.32E-04	-0.021	1.48E-07
20	rs2145270	6,621,685	*BMP2 (a)*	-0.032	7.14E-08	-0.016	6.10E-03	0.027	8.82E-06	-0.030	3.44E-14	-0.019	1.97E-06	0.030	6.91E-14
20	rs143384	34,025,756	*GDF5 (a)*	-0.014	2.07E-02	-0.045	2.27E-14	0.050	5.16E-17	-0.012	2.83E-03	-0.044	5.92E-28	0.047	7.71E-33

* - The 'clump' function in PLINK was used to identify signals for association that were independent in each GWAS (see methods). Several the regions were associated with more than one of the three body fat ratios or associated with one body fat ratio in more than one of the strata (males, females or sex-combined). As a result, different leading SNPs were observed for different traits and strata at several loci. To assess whether these represented the same signal, we assessed the LD between the leading SNPs for different traits. SNPs in low LD with the most strongly associated SNP at each locus (R2-value < 0.05) were considered to represent independent signals. The 'Locus' column denotes: (a) the reported gene from previous GWAS, (b) the most proximal gene, (c) the most strongly associated gene that is regulated by an eQTL in LD with the leading SNP, or (d) a gene containing a missense variant in LD with the leading SNP. Direction - summary of effect direction for the discovery and replication cohorts, with one '+' or '-' per cohort. All gene names are in italic font. Further data as well as annotations are available in Supplementary Data 1.

Supplementary Table 5. Independent signals* for association with body fat ratios in males.

				Discovery						Replication					
**CHR**	**RSID**	**BP**	**Locus name**	β_{AFR}	P_{AFR}	β_{LFR}	P_{LFR}	β_{TFR}	P_{TFR}	β_{AFR}	P_{AFR}	β_{LFR}	P_{LFR}	β_{TFR}	P_{TFR}
1	rs4846204	10,308,958	*KIF1B (b)*	-0.007	4.41E-01	-0.028	2.94E-03	0.030	1.03E-03	-0.013	4.63E-02	-0.024	2.21E-04	0.027	2.82E-05
1	rs55750792	17,306,870	*MFAP2 (a)*	-0.002	7.93E-01	0.013	4.00E-02	-0.012	4.78E-02	0.002	5.59E-01	0.005	2.82E-01	-0.006	1.35E-01
1	rs2273368	113,063,771	*WNT2B (a)*	0.010	2.01E-01	-0.002	7.56E-01	-0.001	9.01E-01	0.001	7.89E-01	-0.008	1.28E-01	0.008	1.52E-01
1	rs11205303	149,906,413	*SF3B4 (a)*	-0.014	2.76E-02	-0.030	1.97E-06	0.033	9.70E-08	0.004	3.97E-01	-0.038	1.77E-18	0.035	5.23E-16
1	rs4971091	155,143,768	*KRTCAP2 (b)*	-0.016	1.20E-02	0.008	2.13E-01	-0.002	7.49E-01	-0.012	4.84E-03	0.003	4.66E-01	0.000	9.97E-01
1	rs180921974	155,268,131	*THBS3(d)*	0.036	8.13E-02	0.092	8.95E-06	-0.104	6.51E-07	0.020	1.68E-01	0.083	6.79E-09	-0.090	3.49E-10
1	rs545608	177,899,121	*SEC16B (a)*	0.021	6.88E-03	-0.014	7.52E-02	0.005	5.15E-01	0.027	2.78E-07	0.002	7.53E-01	-0.011	3.63E-02
1	rs5779197	184,009,826	*TSEN15, GLT25D2 (a)*	-0.003	6.45E-01	-0.015	1.97E-02	0.016	1.58E-02	-0.010	2.81E-02	0.002	6.82E-01	0.001	8.51E-01
1	rs991967	218,615,451	*TGFB2 (a)*	0.010	1.40E-01	-0.011	1.21E-01	0.006	3.59E-01	0.002	6.65E-01	0.000	9.80E-01	-0.001	7.60E-01
1	rs2820443	219,753,509	*LYPLAL1 (a)*	0.003	6.25E-01	-0.006	3.48E-01	0.004	5.28E-01	-0.001	8.03E-01	-0.010	2.40E-02	0.010	3.28E-02
1	rs10916174	227,804,041	*ZNF678 (a)*	0.015	7.39E-02	-0.006	4.82E-01	0.001	8.67E-01	0.004	5.31E-01	-0.007	2.29E-01	0.006	2.83E-01
2	rs62107261	422,144	*FAM150B (a)*	-0.042	0.003548	0.003	0.8195	0.014	0.3249	-0.055	3.03E-08	0.016	1.05E-01	0.006	5.26E-01
2	rs6731872	624,205	*TMEM18 (a)*	-0.040	1.01E-06	-0.012	1.59E-01	0.026	1.36E-03	-0.031	3.26E-08	-0.009	1.08E-01	0.019	5.77E-04
2	rs754537	25,176,277	*RBJ, DNAJC27 (a)*	0.000	9.83E-01	0.002	7.68E-01	-0.001	8.45E-01	0.000	9.20E-01	0.001	8.80E-01	0.000	9.72E-01
2	rs17511102	37,960,613	*CDC42EP3 (a)*	-0.002	8.23E-01	-0.038	4.37E-04	0.039	2.61E-04	0.009	2.40E-01	-0.013	7.56E-02	0.009	2.24E-01
2	rs7562173	46,976,217	*SOCS5 (a)*	0.008	2.22E-01	0.001	8.63E-01	-0.002	7.77E-01	0.002	6.85E-01	-0.005	2.61E-01	0.005	2.72E-01
2	rs3791679	56,096,892	*EFEMP1 (a)*	-0.010	1.72E-01	0.008	2.81E-01	-0.002	7.45E-01	-0.008	1.36E-01	0.018	5.79E-04	-0.015	3.86E-03
2	rs13011472	57,961,602	*VRK2 (b)*	-0.021	6.53E-04	0.011	7.94E-02	-0.003	6.13E-01	-0.011	8.25E-03	0.001	8.19E-01	0.004	3.35E-01
2	rs148812496	198,540,352	*RFTN (b)*	-0.029	2.41E-04	-0.019	1.36E-02	0.029	2.10E-04	-0.012	2.10E-02	-0.011	3.24E-02	0.016	2.33E-03
3	rs4521268	49,137,904	*WDR6(c)*	0.001	9.35E-01	0.017	7.76E-03	-0.016	1.64E-02	0.000	9.74E-01	0.016	3.27E-04	-0.017	1.86E-04
3	rs1986599	50,034,637	*RBM6 (b)*	0.002	8.67E-01	0.030	2.80E-03	-0.031	1.85E-03	0.019	3.77E-03	0.017	1.05E-02	-0.024	1.66E-04
3	rs13069365	51,016,959	*DOCK3 (a)*	-0.041	1.00E-05	0.003	7.85E-01	0.012	1.83E-01	-0.015	1.51E-02	-0.007	2.50E-01	0.014	3.20E-02
3	rs565817433	52,718,183	*NEK4(c)*	0.005	4.07E-01	0.020	1.54E-03	-0.023	1.88E-04	0.006	1.62E-01	0.003	4.75E-01	-0.004	3.15E-01
3	rs9853018	141,101,961	*ZBTB38 (a)*	0.014	2.59E-02	-0.003	6.43E-01	-0.001	8.27E-01	0.015	3.56E-04	0.001	8.84E-01	-0.006	1.33E-01

4	rs2241069	8,602,798	*CPZ (b)*	-0.006	3.36E-01	-0.004	5.30E-01	0.007	2.84E-01	-0.001	8.03E-01	-0.010	2.08E-02	0.009	2.90E-02
4	rs79334166	17,859,466	*FAM184B, C4orf30 (a)*	-0.023	1.16E-02	0.005	5.64E-01	0.003	7.55E-01	-0.030	2.55E-06	0.020	1.43E-03	-0.009	1.51E-01
4	rs4694504	73,496,691	*ADAMTS3 (a, c)*	-0.011	6.66E-02	-0.006	3.43E-01	0.009	1.50E-01	-0.003	4.15E-01	-0.016	1.84E-04	0.017	5.14E-05
4	rs994014	82,165,790	*PRKG2 (a)*	-0.005	4.89E-01	0.004	5.78E-01	-0.003	6.80E-01	-0.010	2.64E-02	-0.006	1.97E-01	0.008	9.73E-02
4	rs7680661	145,565,116	*HHIP (a)*	-0.001	8.94E-01	0.016	5.03E-02	-0.016	5.74E-02	0.001	9.02E-01	0.010	7.25E-02	-0.010	8.82E-02
5	rs465983	55,812,130	*MAP3K1 (a)*	0.008	2.66E-01	-0.015	3.57E-02	0.013	7.14E-02	-0.001	8.89E-01	-0.017	7.19E-04	0.017	5.66E-04
5	rs34341	74,934,009	*ANKDD1B(c)*	-0.011	7.99E-02	-0.002	8.01E-01	0.004	4.98E-01	-0.005	2.43E-01	-0.008	5.24E-02	0.011	9.57E-03
5	rs115912456	82,815,158	*VCAN (a)*	0.069	8.19E-06	0.042	7.13E-03	-0.061	7.67E-05	0.026	1.68E-02	0.013	2.19E-01	-0.022	3.84E-02
5	rs3812049	127,418,850	*SLC12A2 (b)*	0.061	9.24E-18	-0.049	4.15E-12	0.026	2.36E-04	0.055	1.15E-29	-0.035	1.38E-12	0.014	3.42E-03
5	rs1317415	157,952,404	*RP11-32D16.1 (b)*	-0.007	3.06E-01	0.002	7.82E-01	0.001	9.22E-01	0.006	2.08E-01	-0.011	2.25E-02	0.009	5.12E-02
5	rs351855	176,520,243	*FGFR4 (a)*	0.034	4.24E-07	-0.012	7.72E-02	0.001	9.22E-01	0.021	8.00E-06	-0.001	7.63E-01	-0.006	2.02E-01
5	rs34022431	176,677,563	*NSD1 (b)*	0.062	1.56E-03	-0.002	9.16E-01	-0.018	3.64E-01	0.018	1.70E-01	0.015	2.54E-01	-0.020	1.34E-01
5	rs888762	178,547,313	*ADAMTS2 (b)*	-0.010	1.26E-01	-0.012	6.69E-02	0.015	2.21E-02	0.001	8.99E-01	-0.011	1.88E-02	0.011	1.50E-02
6	rs41271299	19,839,415	*ID4 (a)*	0.009	5.31E-01	0.001	9.49E-01	-0.001	9.53E-01	0.002	8.72E-01	0.003	7.73E-01	-0.005	6.31E-01
6	rs9358913	26,239,404	*HIST1H2BE (a)*	-0.005	4.81E-01	-0.003	7.18E-01	0.005	4.88E-01	0.014	4.07E-03	0.003	5.53E-01	-0.006	2.05E-01
6	rs9469762	34,236,430	*HMGA1 (a)*	0.016	4.05E-01	-0.001	9.42E-01	-0.005	8.12E-01	0.021	9.68E-02	0.023	8.02E-02	-0.028	3.16E-02
6	rs2492863	34,603,646	*C6orf106 (b)*	0.007	4.53E-01	-0.014	1.12E-01	0.011	2.24E-01	0.014	2.31E-02	0.000	9.77E-01	-0.005	3.88E-01
6	rs2635727	50,820,940	*TFAP2 (a)*	-0.027	1.92E-04	0.005	4.68E-01	0.004	5.84E-01	-0.019	1.28E-04	0.013	9.34E-03	-0.004	4.25E-01
6	rs314263	105,392,745	*LIN28B (a)*	-0.004	5.75E-01	-0.025	1.33E-04	0.026	6.94E-05	-0.012	8.17E-03	-0.031	1.94E-11	0.033	2.36E-13
6	rs6570507	142,679,572	*GPR126 (a)*	-0.004	5.37E-01	0.012	7.36E-02	-0.010	1.45E-01	0.004	4.25E-01	0.017	2.71E-04	-0.019	3.88E-05
6	rs2982708	152,356,220	*ESR1 (a)*	-0.005	4.32E-01	0.010	1.56E-01	-0.006	3.75E-01	0.001	9.06E-01	-0.009	5.40E-02	0.009	5.44E-02
7	rs798491	2,800,521	*AMZ1 (a)*	-0.011	9.18E-02	0.023	5.88E-04	-0.016	1.48E-02	-0.014	3.19E-03	0.021	6.18E-06	-0.016	5.70E-04
7	rs3823974	20,442,796	*ITGB8 (b)*	-0.003	6.04E-01	0.001	8.47E-01	0.000	9.38E-01	0.006	2.03E-01	0.005	2.21E-01	-0.006	1.85E-01
7	rs481806	28,207,300	*JAZF1 (a)*	-0.019	3.91E-03	-0.003	6.31E-01	0.010	1.35E-01	-0.004	3.53E-01	-0.009	5.46E-02	0.010	3.72E-02
7	rs527582137	92,251,321	*CDK6 (a)*	0.008	2.39E-01	0.010	1.59E-01	-0.013	7.20E-02	0.011	3.15E-02	-0.011	3.12E-02	0.007	1.61E-01
7	rs112416074	148,647,760	*PDIA4 (a)*	0.002	8.05E-01	-0.019	2.25E-02	0.016	5.41E-02	0.005	4.14E-01	-0.008	1.55E-01	0.006	2.55E-01
7	rs56282717	150,657,095	*KCNH2 (b)*	-0.031	2.33E-05	0.003	7.10E-01	0.008	3.02E-01	-0.017	8.27E-04	-0.002	6.66E-01	0.009	8.62E-02

8	rs2044387	8,907,950	*ERI1 (b)*	0.000	9.62E-01	0.000	9.69E-01	0.000	9.70E-01	0.010	2.32E-02	-0.002	6.23E-01	-0.002	6.62E-01
8	rs12546366	10,802,146	*XKR6 (b)*	0.005	3.83E-01	0.005	4.23E-01	-0.005	4.12E-01	0.018	2.46E-05	0.009	2.80E-02	-0.015	4.05E-04
8	rs4733727	130,731,484	*MLZE, GSDMC (a)*	-0.005	3.88E-01	0.004	4.90E-01	-0.002	7.02E-01	-0.006	1.36E-01	0.007	1.11E-01	-0.003	4.21E-01
9	rs10962638	16,846,111	*BNC2 (b)*	0.008	3.85E-01	0.021	1.87E-02	-0.024	8.75E-03	0.000	9.90E-01	0.008	1.59E-01	-0.008	1.84E-01
9	rs35344761	78,510,823	*PCSK5 (a)*	-0.003	7.17E-01	0.020	3.79E-02	-0.016	9.20E-02	0.006	3.61E-01	0.006	3.30E-01	-0.010	1.44E-01
9	rs7039458	86,639,999	*RMI1 (c,d)*	0.019	8.65E-03	-0.016	2.94E-02	0.010	1.84E-01	0.003	5.31E-01	-0.021	1.67E-05	0.020	6.07E-05
9	rs3780327	129,945,847	*RALGPS1 (b)*	0.003	6.90E-01	-0.035	4.33E-06	0.032	2.39E-05	0.005	3.32E-01	-0.022	2.20E-05	0.020	1.09E-04
10	rs34821335	72,433,203	*ADAMTS14(c)*	-0.019	7.45E-03	0.008	2.64E-01	-0.001	8.39E-01	-0.013	7.91E-03	-0.005	3.29E-01	0.009	7.38E-02
10	rs11289753	96,026,184	*PLCE1 (b)*	0.042	1.10E-11	-0.004	5.52E-01	-0.009	1.32E-01	0.033	1.71E-14	-0.006	1.59E-01	-0.006	1.92E-01
11	rs1138714	825,110	*AP006621.1 (b)*	-0.023	2.63E-04	0.004	5.55E-01	0.004	5.68E-01	-0.010	1.82E-02	0.001	7.67E-01	0.003	4.55E-01
11	rs10769282	47,626,492	*MTCH2 (a)*	-0.018	8.53E-03	0.025	2.87E-04	-0.017	1.36E-02	-0.015	9.87E-04	0.019	5.02E-05	-0.012	7.71E-03
11	rs71455793	65,715,204	*TSGA10IP(d)*	0.023	1.20E-01	0.022	1.35E-01	-0.024	1.10E-01	0.020	4.74E-02	-0.009	3.71E-01	0.006	5.65E-01
11	rs552846225	66,913,469	*KDM2A (a)*	0.007	5.12E-01	0.031	2.79E-03	-0.032	2.81E-03	0.002	7.97E-01	0.009	2.06E-01	-0.008	2.60E-01
11	rs4980661	69,306,579	*MYEOV, CCND1 (a)*	0.021	7.50E-04	-0.010	1.12E-01	0.003	6.58E-01	0.013	1.77E-03	-0.001	7.54E-01	-0.004	4.08E-01
11	rs1789166	69,482,091	*ORAOV1 (b)*	-0.020	2.01E-03	0.013	5.25E-02	-0.004	4.90E-01	-0.011	1.20E-02	0.006	1.94E-01	-0.002	6.10E-01
12	rs34716573	576,037	*B4GALNT3 (a)*	0.001	8.40E-01	-0.006	3.79E-01	0.007	2.89E-01	0.004	3.82E-01	-0.001	8.23E-01	-0.001	7.53E-01
12	rs11049566	28,532,961	*CCDC91 (a)*	0.014	4.11E-02	0.010	1.28E-01	-0.013	5.48E-02	0.009	4.53E-02	0.003	5.43E-01	-0.006	1.85E-01
12	rs7306275	50,250,111	*BCDIN3D, FAIM2 (a)*	0.015	1.85E-02	-0.012	5.68E-02	0.007	3.03E-01	0.014	2.07E-03	-0.004	3.18E-01	-0.001	7.87E-01
12	rs11614785	50,880,422	*LARP4 (b)*	-0.015	2.58E-02	0.009	1.63E-01	-0.003	6.06E-01	-0.009	4.60E-02	0.004	3.38E-01	-0.002	6.27E-01
12	rs6489111	123,051,018	*KNTC1 (a)*	0.001	9.18E-01	-0.006	3.36E-01	0.005	4.30E-01	0.014	1.85E-03	-0.001	7.95E-01	-0.004	3.27E-01
13	rs6492538	91,993,746	*GPC5 (b)*	0.000	9.92E-01	-0.001	9.46E-01	-0.002	8.27E-01	-0.003	5.71E-01	0.003	5.46E-01	-0.003	5.87E-01
14	rs71420186	50,960,918	*MAP4K5 (b)*	0.035	6.04E-03	-0.056	9.99E-06	0.042	8.35E-04	0.014	1.15E-01	-0.045	1.82E-07	0.038	8.79E-06
15	rs35874463	67,457,698	*SMAD3(d)*	0.003	8.36E-01	-0.005	6.74E-01	0.007	6.13E-01	0.008	3.99E-01	-0.035	1.42E-04	0.028	2.37E-03
15	rs12905253	74,232,437	*PML (a)*	0.007	2.89E-01	0.004	5.09E-01	-0.005	4.09E-01	0.011	8.21E-03	0.008	7.02E-02	-0.011	1.21E-02
15	rs11856122	84,576,348	*ADAMTSL3 (a)*	0.031	4.29E-07	-0.012	5.48E-02	-0.001	9.27E-01	0.026	4.98E-10	-0.003	5.48E-01	-0.006	1.64E-01
15	rs11856122	89,361,669	*RP11-343B18.2(c)*	0.000	9.68E-01	-0.007	2.57E-01	0.006	3.44E-01	0.004	3.56E-01	-0.011	8.22E-03	0.010	2.32E-02

15	rs28584580	89,397,827	*ACAN (a)*	0.006	7.27E-01	-0.044	1.76E-02	0.039	3.56E-02	0.018	1.50E-01	-0.017	1.72E-01	0.013	2.83E-01
15	rs3817428	89,415,247	*ACAN (a)*	0.013	6.61E-02	0.007	2.89E-01	-0.012	8.44E-02	0.025	1.74E-07	0.004	3.56E-01	-0.012	9.82E-03
15	rs72755233	100,692,953	*ADAMTS17(d)*	0.022	2.29E-02	0.002	8.38E-01	-0.010	3.24E-01	0.018	6.86E-03	0.015	2.86E-02	-0.021	1.83E-03
15	rs4988781	100,800,840	*ADAMTS17 (a)*	0.001	8.60E-01	0.000	9.50E-01	0.000	9.85E-01	0.004	3.93E-01	-0.011	1.32E-02	0.009	4.68E-02
16	rs369515117	28,862,780	*SH2B1 (a)*	0.012	6.09E-02	-0.014	3.68E-02	0.008	1.98E-01	0.015	7.33E-04	-0.019	2.09E-05	0.013	4.26E-03
16	rs8050894	31,104,509	*KAT8(c)*	-0.025	9.78E-05	0.002	7.88E-01	0.005	4.03E-01	-0.006	1.65E-01	0.003	5.15E-01	-0.001	8.00E-01
16	rs55872725	53,809,123	*FTO(a)*	0.045	9.81E-13	-0.002	7.29E-01	-0.016	1.21E-02	0.036	1.39E-16	0.005	2.70E-01	-0.021	1.26E-06
16	rs8057620	69,884,619	*RP11-419C5.2(c)*	-0.008	1.93E-01	-0.022	3.64E-04	0.022	3.97E-04	0.009	2.65E-02	-0.014	1.38E-03	0.010	1.78E-02
16	rs10584116	90,062,323	*C16orf3(c)*	0.009	4.11E-01	0.002	8.42E-01	-0.005	6.04E-01	0.014	4.45E-02	-0.002	7.64E-01	-0.001	9.01E-01
17	rs2071167	42,287,519	*ASB16(c)*	-0.014	5.93E-02	-0.025	4.52E-04	0.029	6.80E-05	-0.003	5.01E-01	-0.002	7.03E-01	0.003	5.41E-01
17	rs2074188	45,888,251	*SP6, SP2 (a)*	0.000	9.88E-01	-0.019	2.17E-03	0.018	3.09E-03	-0.006	1.73E-01	0.001	7.93E-01	0.001	7.30E-01
17	rs28394864	47,450,775	*ZNF652 (b)*	0.004	5.09E-01	0.006	2.92E-01	-0.007	2.37E-01	0.019	1.49E-05	0.007	9.58E-02	-0.012	6.58E-03
18	rs4800148	20,724,328	*RBBP8, CABLES1, C18orf45 (a)*	-0.003	7.14E-01	-0.011	1.30E-01	0.012	1.15E-01	0.008	1.19E-01	-0.009	8.32E-02	0.006	2.48E-01
18	rs7236575	46,653,380	*DYM (a)*	-0.004	6.51E-01	0.031	5.34E-04	-0.030	7.15E-04	-0.001	8.34E-01	0.005	4.65E-01	-0.005	4.30E-01
18	rs71336392	57,850,927	*MC4R (a)*	0.037	4.54E-07	0.001	8.66E-01	-0.017	1.87E-02	0.048	8.48E-22	-0.003	5.80E-01	-0.014	6.21E-03
19	rs62621197	8,670,147	*ADAMTS10(d)*	0.007	7.16E-01	0.028	1.33E-01	-0.029	1.22E-01	0.007	5.65E-01	0.037	2.75E-03	-0.036	3.03E-03
19	rs10402308	19,657,500	*GATAD2A (a)*	-0.027	9.45E-04	0.036	7.06E-06	-0.026	1.22E-03	-0.016	3.75E-03	0.017	1.71E-03	-0.013	1.79E-02
19	rs12971970	47,559,273	*ZC3H4 (a)*	-0.025	4.69E-04	0.004	5.37E-01	0.005	5.24E-01	-0.015	2.17E-03	-0.012	1.97E-02	0.017	5.58E-04
20	rs6085551	6,408,920	*RP11-199O14.1 (b)*	0.035	2.18E-08	-0.004	5.18E-01	-0.005	3.80E-01	0.011	1.14E-02	-0.002	7.22E-01	-0.001	8.47E-01
20	rs2145270	6,621,685	*BMP2 (a)*	-0.022	7.20E-04	0.008	2.29E-01	0.001	9.10E-01	-0.009	4.33E-02	0.008	5.38E-02	-0.006	1.96E-01
20	rs143384	34,025,756	*GDF5 (a)*	0.035	1.34E-08	-0.029	3.25E-06	0.017	0.01	0.025	1.29E-08	-0.017	7.22E-05	0.009	4.48E-02

* - The 'clump' function in PLINK was used to identify signals for association that were independent in each GWAS (see methods). Several the regions were associated with more than one of the three body fat ratios or associated with one body fat ratio in more than one of the strata (males, females or sex-combined). As a result, different leading SNPs were observed for different traits and strata at several loci. To assess whether these represented the same signal, we assessed the LD between the leading SNPs for different traits. SNPs in low LD with the most strongly associated SNP at each locus (R2-value < 0.05) were considered to represent independent signals. The 'Locus' column denotes: (a) the reported gene from previous GWAS, (b) the most proximal gene, (c) the most strongly associated gene that is regulated by an eQTL in LD with the leading SNP, or (d) a gene containing a missense variant in LD with the leading SNP. Direction - summary of effect direction for the discovery and replication cohorts, with one '+' or '-' per cohort. All gene names are in italic font. Further data as well as annotations are available in Supplementary Data 1.

Supplementary Table 6. Direction of effect for body fat ratio–associated lead SNPs for standard anthropometric traits in the UK biobank cohort. CHR – chromosome. BP – position in base pairs of lead SNP, AFR – Arm fat ratio, LFR – leg fat ratio, TFR – trunk fat ratio, – BMI – body mass index, WC – waist circumference, WHR – Waist–to–hip ratio, WCadjBMI – waist circumference adjusted for BMI, WHRadjBMI – waist–to–hip ratio adjusted for BMI. '+' – the direction of effect of the minor allele is positive. '- ' – the direction of effect of the minor allele is negative. Direction of effects are shown for all traits that were associated ($P < 1*10^{-7}$) with the SNPs.

Phenotype in previous GWAS	SNP	CHR	BP	AFR	LFR	TFR	BMI	Height	WC	WHR	WC-adj BMI	WHR-adj BMI
**Appendicular lean mass**	rs115912456	5	82,815,158		-	+						
**Birth weight**	rs2074188	17	45,888,251		-	+						
**Body fat percentage, WC, Obesity, BMI, Weight,**	rs369515117	16	28,862,780		-	+	+			+		
**Height**	rs9853018	3	141,101,961		-	+		+		+		+
**Height**	rs41271299	6	19,839,415		-	+		+				+
**Height, HCadjBMI**	rs991967	1	218,615,451		-	+		+				+
**Height, HCadjBMI**	rs994014	4	82,165,790		-	+		+				+
**Height, HCadjBMI**	rs34716573	12	576,037		-	+		+				+
**Height, HCadjBMI, WCadjBMI**	rs4694504	4	73,496,691		-	+		+				+
**Height, Infant length, Hip circumference, HCadjBMI**	rs143384	20	34,025,756		-	+		+				+
**Height, WCadjBMI**	rs5779197	1	184,009,826		-	+		+				+
**Height, WCadjBMI**	rs314263	6	105,392,745		-	+		+				+
**Infant length, Height, WCadjBMI, HCadjBMI**	rs11205303	1	149,906,413		-	+		+				+
**WHRadjBMI, Hip circumference, HCadjBMI**	rs2820443	1	219,753,509		-	+			-		-	
**Height**	rs17511102	2	37,960,613			+		+				
**Height**	rs13069365	3	51,016,959			+		+				
**Height**	rs2982708	6	152,356,220			+		+				

Phenotype in previous GWAS	SNP	CHR	BP	AFR	LFR	TFR	BMI	Height	WC	WHR	WC-adj BMI	WHR-adj BMI
**Height**	rs112416074	7	148,647,760			+		+				
**Height**	rs4988781	15	100,800,840			+		+				+
**WCadjBMI**	rs481806	7	28,207,300			+		+				+
**WCadjBMI**	rs527582137	7	92,251,321			+		+				
**WHRadjBMI**	rs465983	5	55,812,130			+			+		+	+
**Height**	rs11856122	15	84,576,348	+	+	-		-				-
**Height**	rs10402308	19	19,657,500	-	+	-		-				
**Height**	rs7680661	4	145,565,116		+	-		-				-
**Height**	rs552846225	11	66,913,469		+	-		-				-
**Height**	rs12905253	15	74,232,437		+	-		-				
**Height**	rs28584580	15	89,397,827		+	-		-				-
**Height**	rs7236575	18	46,653,380		+	-		-				
**Height, Infant length, WCadjBMI**	rs3817428	15	89,415,247		+	-		-				-
**Height, WCadjBMI**	rs9358913	6	26,239,404		+	-		-		-		
**Height, WCadjBMI**	rs35344761	9	78,510,823		+	-		-				-
**Height, WCadjBMI**	rs4800148	18	20,724,328		+	-		-		-		-
**Height, WCadjBMI, Birth weight**	rs798491	7	2,800,521		+	-		-				-
**Height, WCadjBMI, HCadjBMI**	rs3791679	2	56,096,892		+	-		-		-		-
**Height, WCadjBMI, HCadjBMI**	rs11049566	12	28,532,961		+	-		-				-
**WCadjBMI**	rs2273368	1	113,063,771		+	-		-				
**Height**	rs351855	5	176,520,243	+		-						-
**Height, WHRadjBMI, WCadjBMI**	rs2145270	20	6,621,685	-		-	-	+			+	+
**Height**	rs10916174	1	227,804,041			-		-				-
**Height**	rs79334166	4	17,859,466			-		-				

Phenotype in previous GWAS	SNP	CHR	BP	AFR	LFR	TFR	BMI	Height	WC	WHR	WC-adj BMI	WHR-adj BMI
**Height**	rs4733727	8	130,731,484			-		-				-
**Height, Waist circumference adjusted for BMI**	rs55750792	1	17,306,870			-		-				-
**Height, WCadjBMI**	rs6570507	6	142,679,572			-		-				-
**BMI**	rs10769282	11	47,626,492		+		-			-		
**BMI**	rs6489111	12	123,051,018		-			+				
**Height**	rs9469762	6	34,236,430		-			+				
**BMI, Body fat percentage, Waist-hip ratio**	rs55872725	16	53,809,123	+			+		+	+		
**Height, BMI, Obesity**	rs71336392	18	57,850,927	+			+	+	+	+		
**Hip circumference, Body mass index**	rs4980661	11	69,306,579	+			+					
**Obesity, Body mass index**	rs7306275	12	50,250,111	+			+			+		
**Obesity, Body mass index, Waist circumference**	rs545608	1	177,899,121	+			+		+	+		
**BMI, Waist circumference, Hip circumference**	rs12971970	19	47,559,273	-			-			-		
**Body mass index**	rs6731872	2	624,205	-			-		-	-		
**Height**	rs754537	2	25,176,277	-			-	+		-		
**Height**	rs7562173	2	46,976,217	-			-	+				
**WHR**	rs2635727	6	50,820,940	-			-			-		

Supplementary Table 7. Overlap between body fat ratio-associated loci with cardiovascular-, and metabolic disease-associations from previous GWAS. Lead SNPs were cross-referenced with GWAS-catalog. LDs between SNPs were calculated in the data from the UK Biobank. SNPs with $R^2 > 0.1$ were considered to possibly represent the same signal.

Ratio-associated SNP	Chr	BP	Locus	Gwas_catalog_snps	Gwas_catalog_trait
rs2273368	1	113,063,771	*WNT2B (a)*	rs17030613, rs12129649, rs112416932	Blood pressure, Diastolic blood pressure, Mean platelet volume, Platelet count, Platelet distribution width, Plateletcrit
rs6731872	2	624,205	*TMEM18 (a)*	rs2867125	Type 2 diabetes
rs754537	2	25,176,277	*RBJ, DNAJC27 (a)*	rs55701159, rs478222	Diastolic blood pressure, Type 1 diabetes
rs148812496	2	198,540,352	***RFTN(b)***	rs1147169	Low high density lipoprotein cholesterol levels
rs1986599	3	50,034,637	*RBM6 (b)*	rs2013208	HDL cholesterol levels
rs13069365	3	51,016,959	*DOCK3 (a)*	rs743757	Diastolic blood pressure
rs465983	5	55,812,130	*MAP3K1 (a)*	rs459193	Type 2 diabetes
rs34341	5	74,934,009	*ANKDD1B(c)*	rs12654264, rs3846662, rs3846663, rs12916, rs6873472, rs4604177, rs5744672, rs10078021	Diastolic blood pressure, LDL cholesterol levels, Total cholesterol levels, Metabolite levels
rs1317415	5	157,952,404	***RP11-32D16.1 (b)***	rs11953630	Diastolic blood pressure, Systolic blood pressure
rs2492863	6	34,603,646	*C6orf106 (b)*	rs2814944, rs9469890, rs2764209, rs2814993, rs4472337	Total cholesterol levels, Coronary artery disease, HDL cholesterol, Plateletcrit
rs2635727	6	50,820,940	*TFAP2(a)*	rs78648104	Systolic blood pressure
rs481806	7	28,207,300	*JAZF1 (a)*	rs849134, rs849135, rs1708302	Type 2 diabetes
rs2044387	8	8,907,950	***ERI1 (b)***	rs7826238	Systolic blood pressure
rs12546366	8	10,802,146	***XKR6 (b)***	rs1821002, rs6601530, rs11776767, rs10107145 , rs7819412	Carotid intima media thickness, Triglycerides, Systolic blood pressure
rs8050894	16	31,104,509	*KAT8(c)*	rs72799341, rs2032915	Diastolic blood pressure, Triglyceride levels

rs55872725	16	53,809,123	*FTO(a)*	rs9940128, rs8050136, rs9939609, rs9936385	Type 2 diabetes, metabolic syndrome
rs28394864	17	47,450,775	***ZNF652 (b)***	rs12940887, rs62076439, rs16948048, rs7502499	Coronary artery disease, Diastolic blood pressure
rs71336392	18	57,850,927	*MC4R (a)*	rs663129, rs663640, rs12967135, rs34633411	C-reactive protein levels or triglyceride levels pleiotropy, Coronary artery disease, HDL cholesterol, C-reactive protein levels or HDL-cholesterol levels pleiotropy
rs10402308	19	19,657,500	*GATAD2A (a)*	rs3794991, rs17216525, rs2304130, rs16996148	Triglycerides, Total cholesterol, LDL cholesterol, Type 2 diabetes
rs143384	20	34,025,756	*GDF5 (a)*	rs6579245, rs2277862, rs7264396, rs6120974	Total cholesterol levels, Triglycerides

*The 'Locus' column denotes: *(a)* the reported gene from previous GWAS, *(b)* the most proximal gene, *(c)* the most strongly associated gene that is regulated by an eQTL in LD with the lead SNP, or *(d)* a gene containing a missense variant in LD with the lead SNP. Loci in bold type designate novel loci that have not been associated with an anthropometric trait in previous GWAS. All gene names are in italic font.

Supplementary Table 8. Phenotypic correlation between body fat ratios and anthropometric traits. Portion of variance in arm fat ratio (AFR), leg fat ratio (LFR) or trunk fat ratio (TFR) (the response variable) that can be explained (squared semi-partial correlation coefficients) by adding either BMI, height, waist-to-hip ratio (WHR), waist circumference (WC), WHR adjusted for BMI (WHRadjBMI), WC adjusted for BMI (WCadjBMI), or one of the other body fat ratios to linear regression models that include age and principal components. Analyses were performed in females and males separately. The signs (+) or (-) is included for all correlations that were significant after Bonferroni correction for the number of tests performed ($P < 0.05/36$) and denote the directions of the effect-estimates.

Females	**BMI**	**Height**	**WHR**	**WC**	**WHRadjBMI**	**WCadjBMI**	**AFR**	**LFR**	**TFR**
Arm fat ratio (AFR)	78.9% (+)	7.3% (-)	15.3% (+)	54.3% (+)	0.1% (-)	0.9% (-)		2.8% (-)	4.8% (-)
Leg fat ratio (LFR)	12.2% (-)	16.0% (-)	4.1% (-)	17.4% (-)	0.2% (-)	5.1% (-)	2.8% (-)		84.0% (-)
Trunk fat ratio (TFR)	0.002%	25.3% (+)	0.2% (+)	1.5% (+)	0.3% (+)	6.8% (+)	4.8% (-)	83.8% (-)	
Males									
Arm fat ratio (AFR)	4.8% (+)	1.1% (+)	0.001%	2.3% (+)	2.6% (-)	0.8% (-)		0.8% (+)	15.1% (-)
Leg fat ratio (LFR)	0.2% (+)	0.7% (-)	0.003%	0.004%	0.2% (-)	1.0% (-)	0.8% (+)		86.2% (-)
Trunk fat ratio (TFR)	1.9% (-)	0.1% (+)	0.002%	0.4% (-)	1.0% (+)	1.6% (+)	15.4% (-)	83.6% (-)	

Supplementary Table 9. Genetic correlations between body fat ratios and anthropometric traits. Genetic correlation (r_g) was estimated by cross-trait LD-score regression[2] using summary statistics from GWAS for body fat ratios. The genetic correlations were squared for ease of comparison with supplementary Table 3. The signs (+) or (-) is included for all correlations that were significant after Bonferroni correction for the number of tests performed ($P < 0.05/36$), and denote if the correlation is positive or negative

Females	**BMI**	**Height**	**WHR**	**WC**	**WHRadjBMI**	**WCadjBMI**	**AFR**	**LFR**	**TFR**
Arm fat ratio (AFR)	79.2% (+)	16% (-)	16.8% (+)	51.8% (+)	0.6% (-)	9% (-)		0.25%	13% (-)
Leg fat ratio (LFR)	9% (-)	42.3% (-)	3.2% (-)	24.0% (-)	0.4%	18.5% (-)	0.25%		82.8% (-)
Trunk fat ratio (TFR)	0.81% (-)	60.8% (+)	0%	2.6% (+)	0.36%	28.1% (+)	13% (-)	82.8% (-)	
Males									
Arm fat ratio (AFR)	13.0% (+)	7.3% (+)	0.5%	10.2% (+)	10.2% (-)	0.4% (-)		10.9% (-)	0.36%
Leg fat ratio (LFR)	3.6% (+)	7.3% (-)	4.4% (+)	0.64%	0.64%	6.25% (-)	10.9% (-)		85% (-)
Trunk fat ratio (TFR)	14.4% (-)	2.9% (+)	7.8% (-)	6.8% (-)	0.25%	7.8% (+)	0.36%	85% (-)	

Supplementary Table 10. Body fat ratio-associated potentially damaging missense variants.

Lead SNP	Chr	Position (bp)	Missense variant	Position (bp)	Distance to lead SNP (bp)	LD w. lead SNP (R2)	Gene	Amino acid substituion	SIFT* score	Polyphen-2* score
rs4733727	8	130,731,484	rs4144738	130,760,850	29,366	0.91	*GSDMC*	Met/Thr	deleterious	probably damaging
rs180921974	1	155,268,131	rs72704117	155,175,089	93,042	0.95	*THBS3*	Ala/Thr	deleterious	possibly damaging
rs3817428	15	89,415,247	lead SNP				*ACAN*	Asp/Glu	deleterious	benign - possibly damaging
rs72755233	15	100,692,953	lead SNP				*ADAMTS17*	Thr/Ile	deleterious	benign
rs115912456	5	82,815,158	rs61749613	82,815,170	12	1.00	*VCAN*	Lys/Glu	tolerated - deleterious	possibly damaging
rs9358913	6	26,239,404	rs7766641	26,184,102	55,302	0.82	*HIST1H2BE*	Gly/Ser	tolerated	probably damaging
rs351855	5	176,520,243	lead SNP				*FGFR4*	Gly/Arg	tolerated	possibly damaging
rs71420186	14	50,960,918	rs12881869	50,923,249	37,669	0.96	*MAP4K5*	Ala/Thr	tolerated	possibly damaging
rs5779197	1	184,009,826	rs2274432	184,020,945	11,119	0.96	*TSEN15*	Gly/Asp	tolerated	benign - possibly damaging
rs143384	20	34,025,756	rs224331	34,022,387	3,369	0.73	*GDF5; GDF5OS*	Ala/Ser; Ala/Glu	tolerated	benign - possibly damaging
rs62621197	19	8,670,147	lead SNP				*ADAMTS10*	Arg/Gln	tolerated	benign - possibly damaging

*Polyphen-2[3] and SIFT[4] scores were extracted from Ensembl (www.ensembl.org). SIFT utilizes protein sequence data to predict the effects of a missense variant while Polyphen-2 also considers protein structure and evolutionary conservation. Polyphen-2 generates a probability that a missense mutation is damaging, while SIFT generates a probability that the amino acid change is tolerated. Polyphen-2 uses a tree-tiered prediction score: 0-0.15 is predicted to be benign, 0.15 to 0.85 possibly damaging and 0.85 -1.0 more confidently predicted to be damaging (i.e., probably damaging). A SIFT score < 0.05 is predicted to be deleterious[4]. Polyphen-2 and SIFT scores are presented as ranges in the cases where they differ due to being part of different transcript variants.

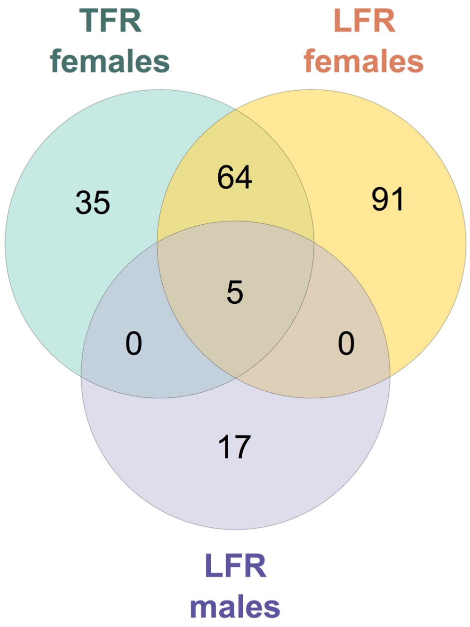

Supplementary Figure 5. Overlap of enriched gene sets from DEPICT for body fat ratios. This figure shows the number of gene sets that were enriched for each body fat ratio in DEPICT analyses and the overlap of gene sets between traits.

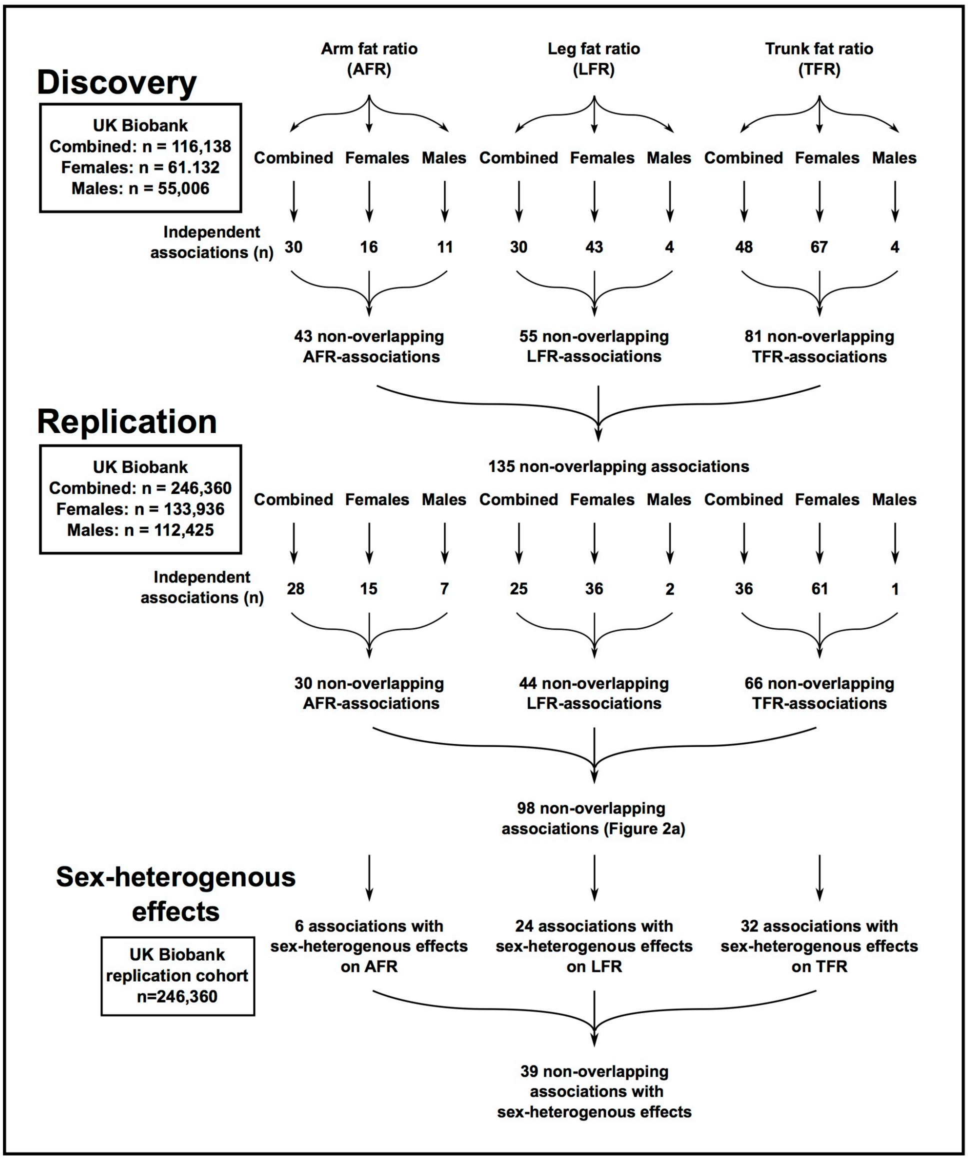

Supplementary Figure 6. Flowchart of the genetic analyses of body-fat ratios. GWAMA was used to detect heterogeneity in genetic effects between sexes[5].

References

1. Bulik-Sullivan, B. *et al.* LD score regression distinguishes confounding from polygenicity in genome-wide association studies. *Nat. Genet.* **47,** 291–295 (2015).
2. Bulik-Sullivan, B. *et al.* An atlas of genetic correlations across human diseases and traits. *Nat. Genet.* **47,** 1236–1241 (2015).
3. Adzhubei, I., Jordan, D. M. & Sunyaev, S. R. *Predicting functional effect of human missense mutations using PolyPhen-2. Current Protocols in Human Genetics* (2013). doi:10.1002/0471142905.hg0720s76
4. Ng, P. C. & Henikoff, S. SIFT : predicting amino acid changes that affect protein function. **31,** 3812–3814 (2003).
5. Magi, R., Lindgren, C. M. & Morris, A. Meta-Analysis of Sex-Specific Genome-Wide Association Studies. *Genet. Epidemiol.* 846–853 (2010). doi:10.1002/gepi.20540
