# Supplementary material for: Genome-wide association study of body fat distribution identifies adiposity loci and sex-specific genetic effects
Source: Nat Commun. 2019 Jan 21;10:339. doi: 10.1038/s41467-018-08000-4 (PMC6341104; doi:10.1038/s41467-018-08000-4)
Supplement: Supplementary file 3 — Description of Additional Supplementary Files [file 41467_2018_8000_MOESM3_ESM.pdf]

Description of Additional Supplementary Files

File Name: Supplementary Data 1

Description: Independent signals* for association with body fat ratios.

File Name: Supplementary Data 2

Description: Sex heterogeneous effects of body fat ratio-associated SNPs was assessed with GWAMA for all replicated trait-associated SNPs. 30 variants were tested for sex-heterogenous effects on AFR, 44 on LFR and 66 on TFR. P-values denote the results from tests for heterogeneity between sexes. Bonferroni correction was used to correct for multiple testing and P-values < 0.05/140 (3.57*10-4) were considered significant. The Locus column denotes a gene related to the associated locus either in annotation from previous GWAS (γ), the most proximal gene (β) or LD (R2>0.8) with a missense variant within the gene (∇ .(All gene names are in italic font.

File Name: Supplementary Data 3

Description: Expression quantitative loci (eQTLS) in LD with leading SNPs at body fat ratio-associated loci.

File Name: Supplementary Data 4

Description: Results from enrichment analyses by DEPICT. Summary stats from sex-stratified GWAS on the combined discovery and replication cohorts were used for DEPICT analyses. The DEPICT protocol uses the 'clump' functionality in PLINK to determine associated loci. The p-value cut off for association for 'clump' was set at P < 10-7. DEPICT determines the enrichment of genes within associated loci in reconstituted gene-sets. These genesets include a membership probability for all genes in the human genome based on similarities in gene expression. FDR was used to adjust for multiple testing. Twelve analyses were run in total (tissue enrichment and gene-set enrichment) and FDR
